# Supplementary material for: Age, Sex, Body Mass Index, Diet and Menopause Related Metabolites in a Large Homogeneous Alpine Cohort
Source: Metabolites. 2022 Feb 24;12(3):205. doi: 10.3390/metabo12030205 (PMC8955763; doi:10.3390/metabo12030205)
Supplement: Supplementary file 1 [file metabolites-12-00205-s001.zip › metabolites-1604168-supplementary/Supplementary_information_v2.pdf]

Supplementary Information for: *Age, sex, body mass index, diet and menopause related metabolites in a large homogenous alpine cohort*

Contents

Data processing and normalization 1

General metabolite overview 2

Signal distribution of individual metabolites 3

Sex-related metabolites 47

Age-related metabolites 49

Metabolites related to body mass index 53

Menopause associated metabolites 55

Metabolites related to food items 57

Seasonality of food items 58

Medication overview 58

Data processing and normalization

Individual metabolite concentrations from study samples were adjusted for between-batch (plate) differences based on concentrations measured in QC samples on the same plate. QC samples considered for normalization were Biocrates QC samples *00p180_QC1* and *00p180_QC2* as well as study-internal QC sample *QC CHRIS Pool* (a pool of all study samples). The effect of the normalization is shown with an relative log abundance (RLA) plot in the figure below.

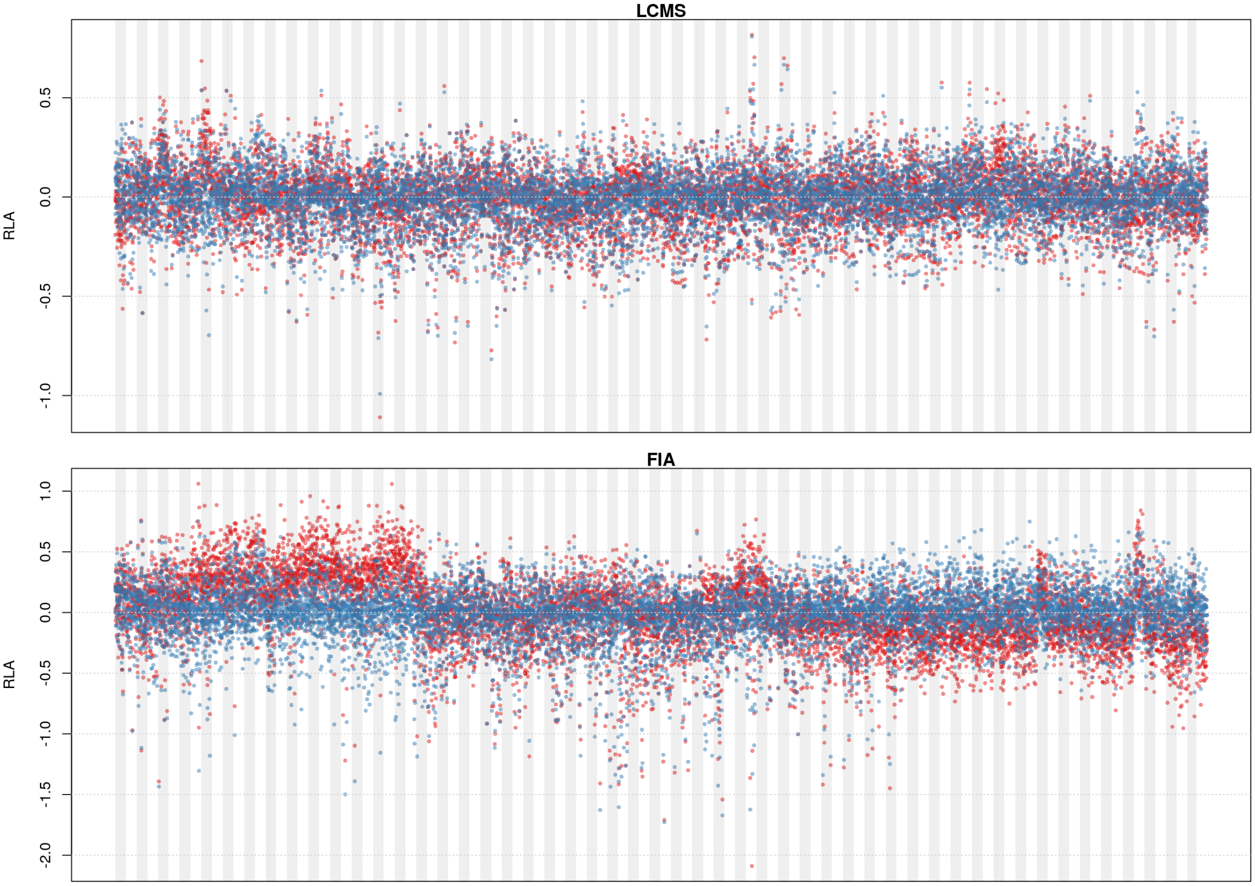

Figure S1: Relative log abundance plots showing the effect of the between-batch normalization for concentrations measured with LC-MS or flow injection analysis (FIA). Each point shows the mean RLA calculated from metabolite concentrations in one study sample. Red and blue points represent the data before and after normalization respectively. Vertical grey-white shading indicates measurements from one plate (batch). Measurements are ordered by date of measurement.

General metabolite overview

Supplementary Table S1 (file *Table_S1-metabolite-ratio-definition.xlsx*): Definition of metabolite ratios.

Supplementary Table S2 (file *Table_S2-metabolite-sum-definition.xlsx*): Definition of groups of metabolites (metabolite sums).

Supplementary Table S3 (file *Table_S3-metabolite-summary.xlsx*): List of all analysed metabolites. *GP*: glycerophospholipids. Columns *Conc* and *Sd* mean and standard deviation of metabolite concentrations (in $\mu mol/L$). *Conc_M* and *Conc_F*: average concentration in male and female participants. *CV_study* and *CV_QC*: coefficient of variation across study or quality control samples (in %). *significant_sex*: for metabolites found to be significantly associated with sex the direction of this association is shown with *pos* and *neg* indicating significantly higher respectively lower concentration in male participants compared to female participants. *significant_age*: for metabolites found to be significantly related to the participants' age the direction of this association is shown (*pos* and *neg* representing increasing or decreasing concentration with increasing age). *significant_BMI*: for metabolite found significantly associated with BMI the direction of this association is shown (*pos* and *neg* representing higher respectively lower concentration in BMI category 4 (obese) against category 2 (normal)). *significant_menopause*: for metabolites significantly related to menopausal the direction of this association is shown with *pos* representing higher concentration in

post-menopausal women. Columns *literature_sex*, *literature_age*, *literature_BMI* and *literature_menopause* contain the Pubmed IDs of the publications in which the metabolite was found significantly related to sex, age, BMI or menopause status. Columns *literature_sex_coef*, *literature_age_coef*, *literature_BMI_coef* and *literature_menopause_coef* indicate whether a positive or negative relationship was found. *Aliases*: alternative name(s) of the metabolite. *Formula*: chemical formula of the metabolite. *HMDB_ID*: identifiers from the Human Metabolom Database annotated to the metabolite.

The xlsx file contains two additional sheets, one with the results from the analysis to identify age, sex and BMI-associated metabolites and one with those from the menopause analysis. Results for all 175 metabolites are reported. Columns *coef*, *p.value*, *p.adj* and *significant* contain the coefficients from the linear regression analysis, the raw and adjusted p-values as well as whether a metabolite was defined to be significant or not for each of the analyzed variables.

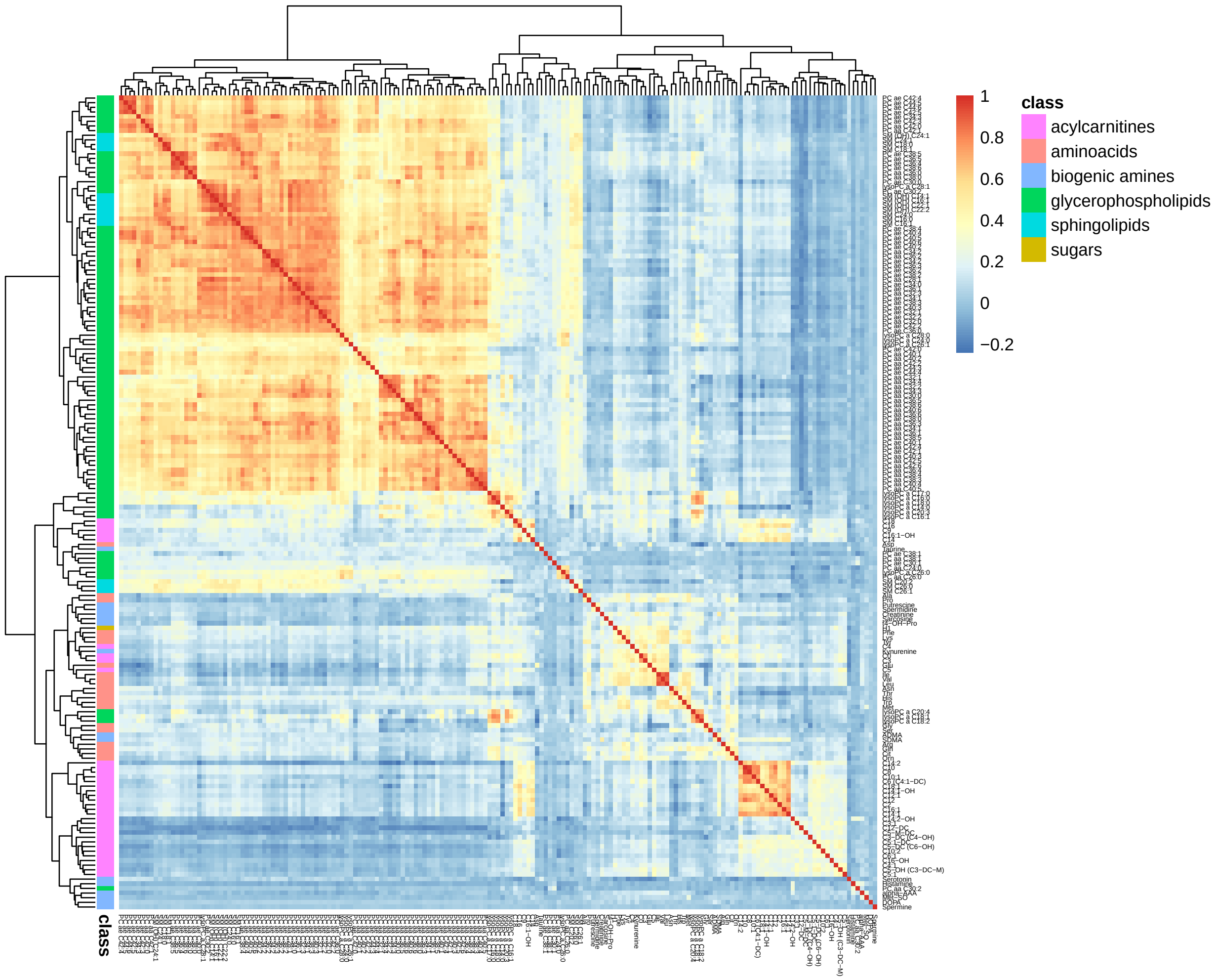

Figure S2: Correlation between metabolite concentrations. Complete linkage hierarchical clustering was performed on pairwise Pearson's correlation coefficients calculated on all study samples.

Signal distribution of individual metabolites

Distribution of log_2 transformed concentrations in analyzed study samples are shown for each analyte. Vertical red lines indicate the *lower level of detection*, blue solid lines the *lower level of quantification* and dashed blue lines the *upper level of quantification* for each metabolite if available.

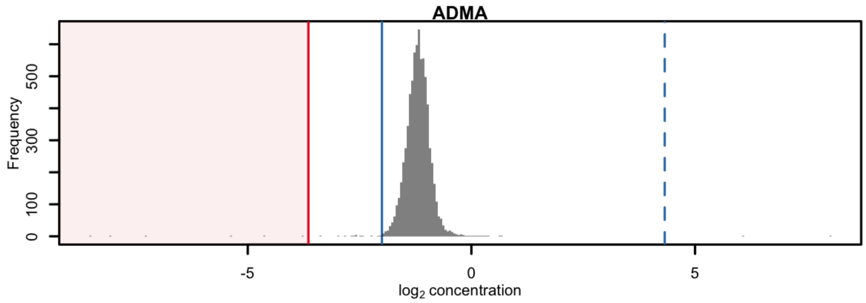

Figure S3: Signal distribution for ADMA.

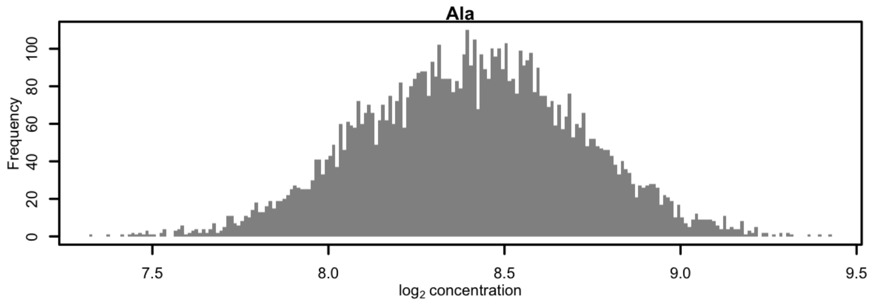

Figure S4: Signal distribution for Ala.

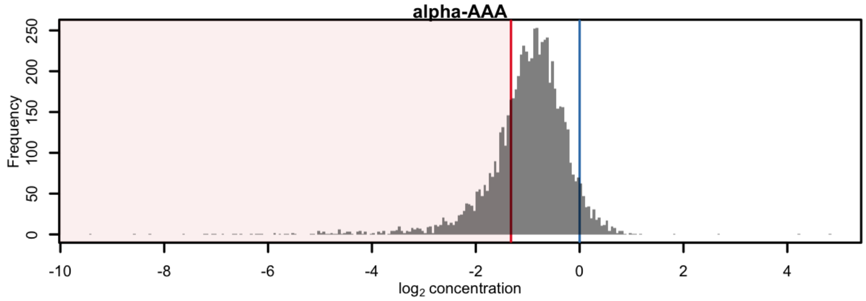

Figure S5: Signal distribution for alpha-AAA.

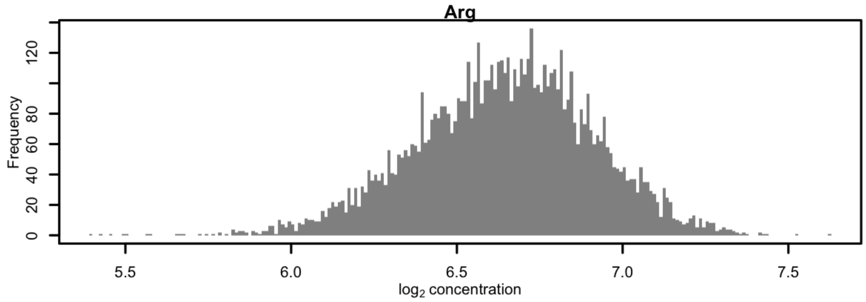

Figure S6: Signal distribution for Arg.

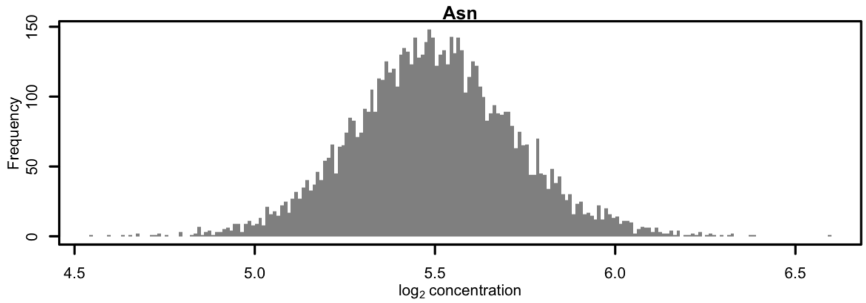

Figure S7: Signal distribution for Asn.

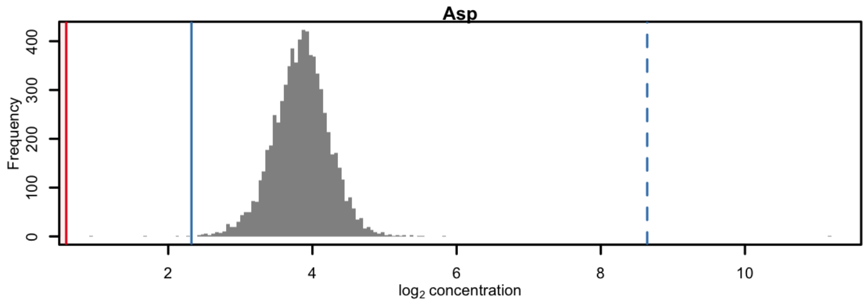

Figure S8: Signal distribution for Asp.

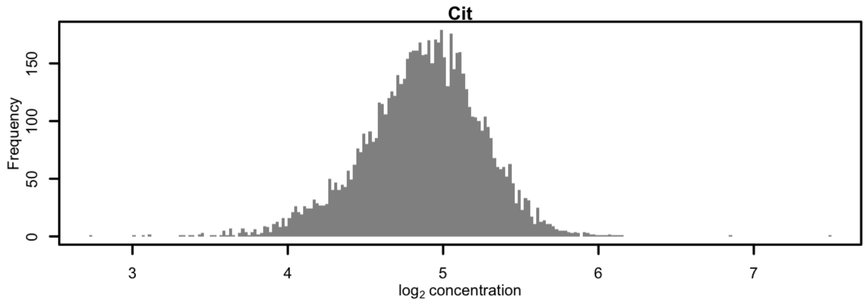

Figure S9: Signal distribution for Cit.

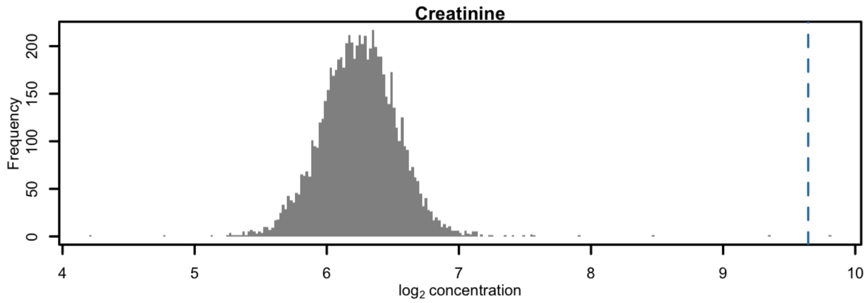

Figure S10: Signal distribution for Creatinine.

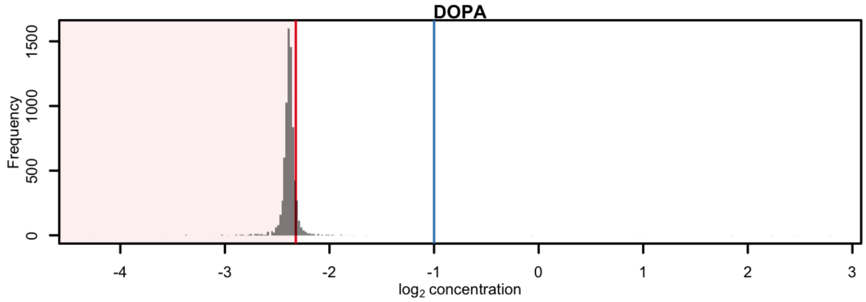

Figure S11: Signal distribution for DOPA.

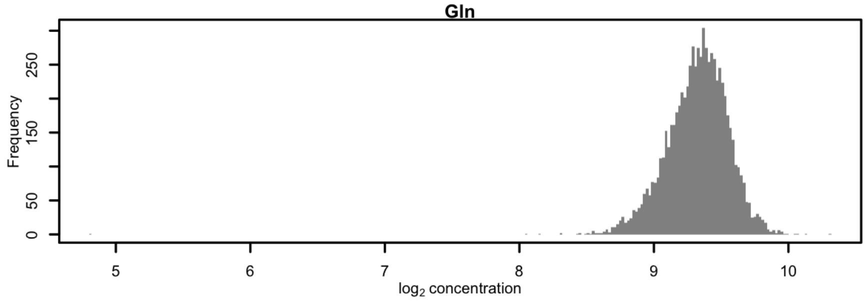

Figure S12: Signal distribution for Gln.

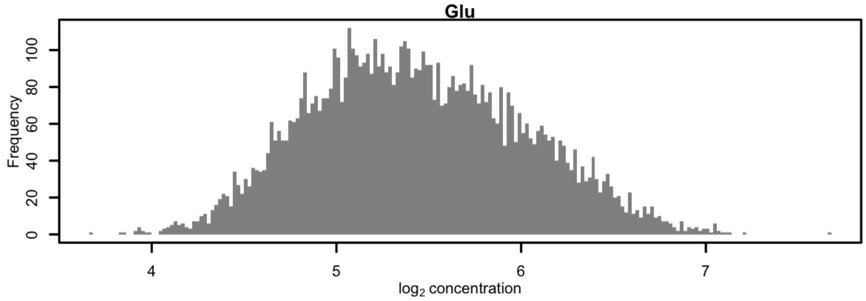

Figure S13: Signal distribution for Glu.

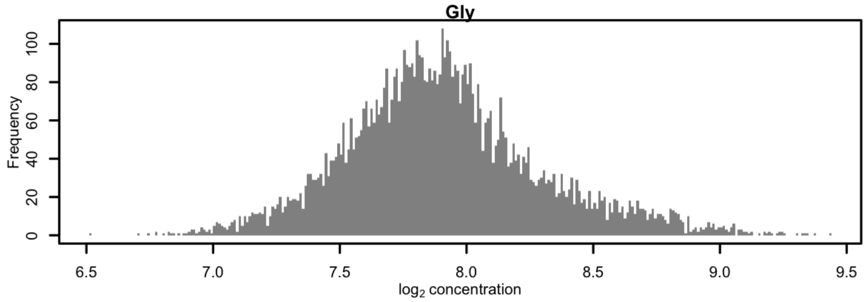

Figure S14: Signal distribution for Gly.

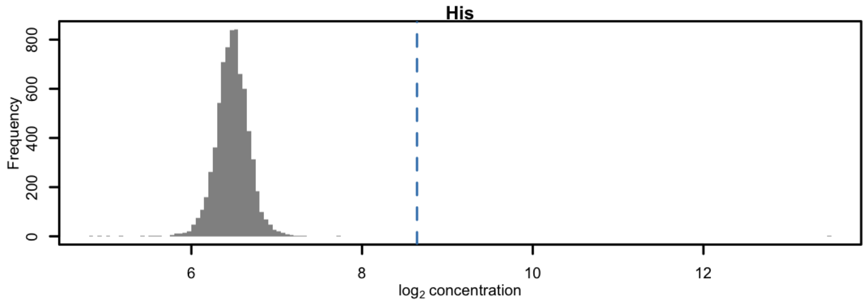

Figure S15: Signal distribution for His.

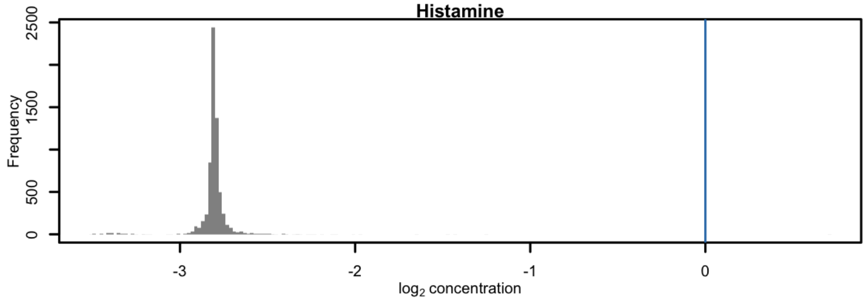

Figure S16: Signal distribution for Histamine.

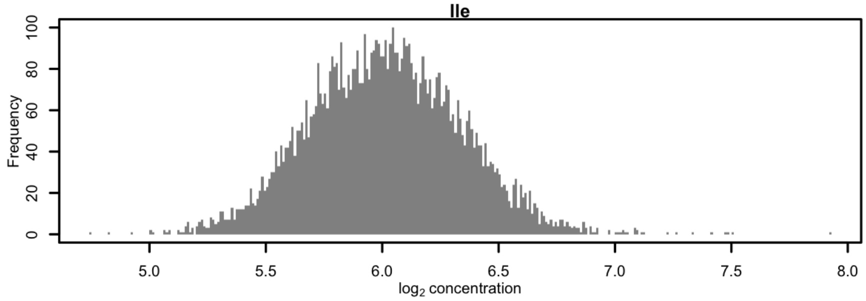

Figure S17: Signal distribution for Ile.

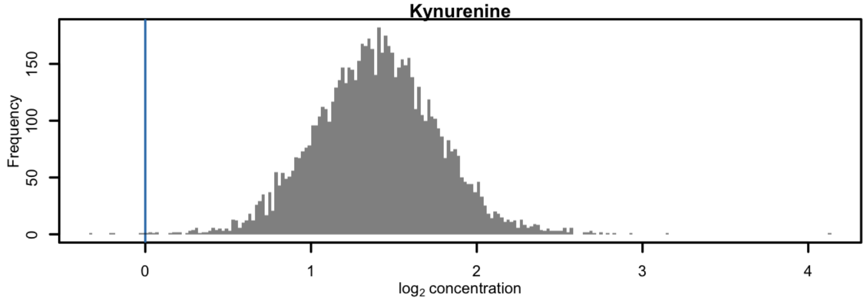

Figure S18: Signal distribution for Kynurenine.

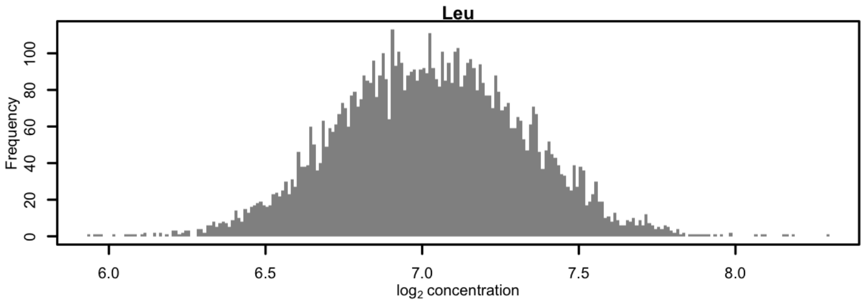

Figure S19: Signal distribution for Leu.

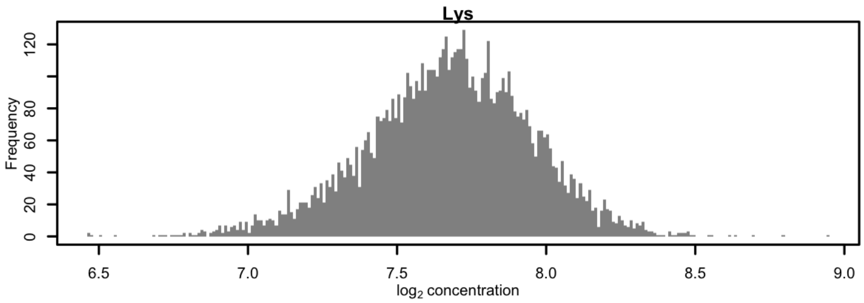

Figure S20: Signal distribution for Lys.

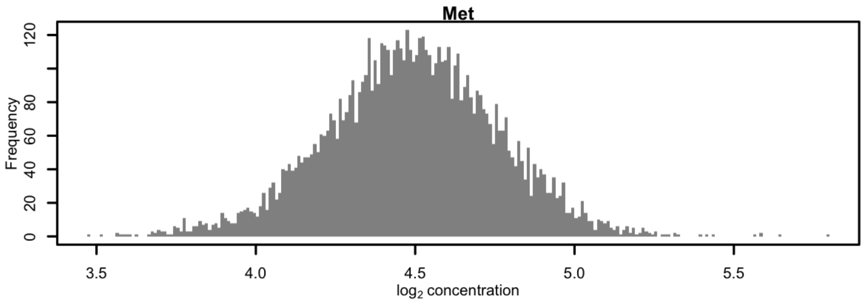

Figure S21: Signal distribution for Met.

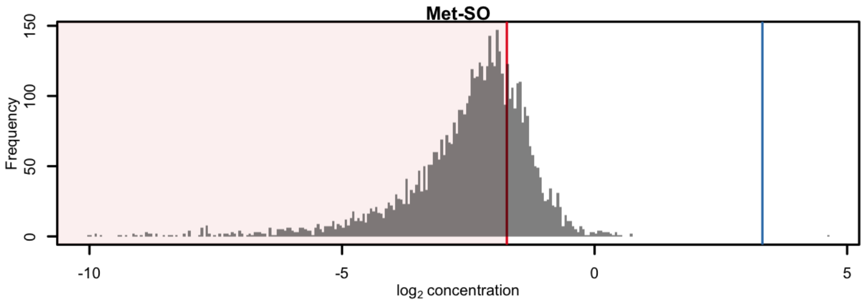

Figure S22: Signal distribution for Met-SO.

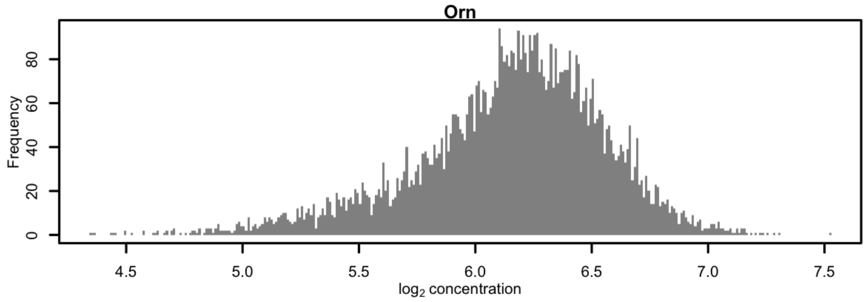

Figure S23: Signal distribution for Orn.

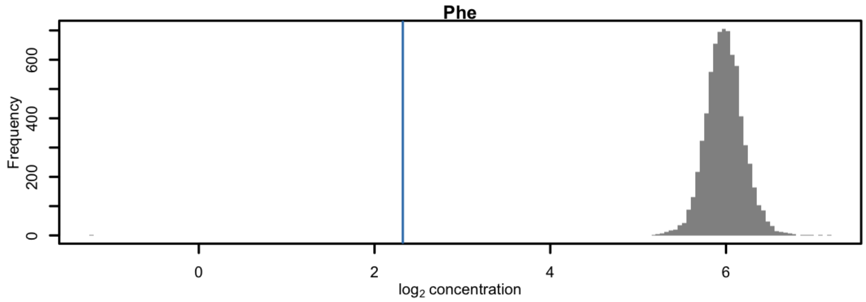

Figure S24: Signal distribution for Phe.

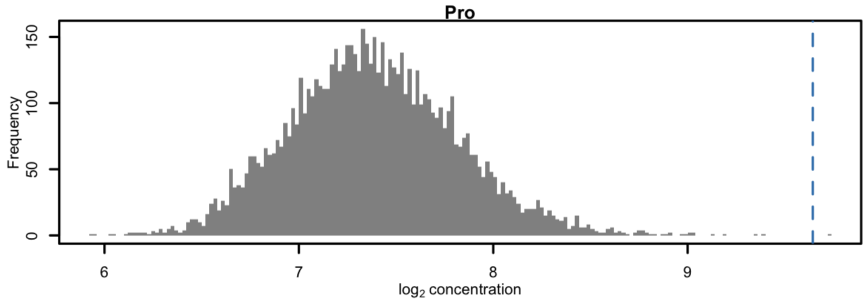

Figure S25: Signal distribution for Pro.

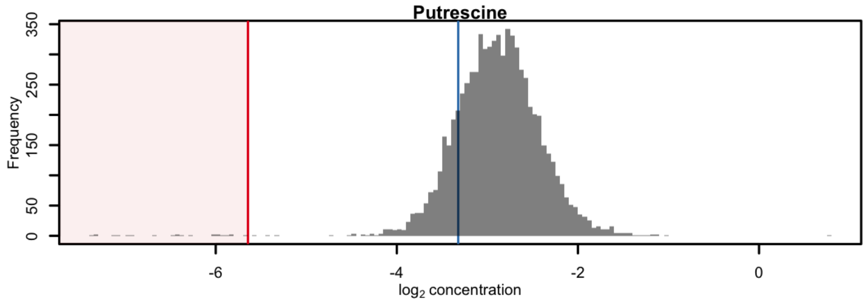

Figure S26: Signal distribution for Putrescine.

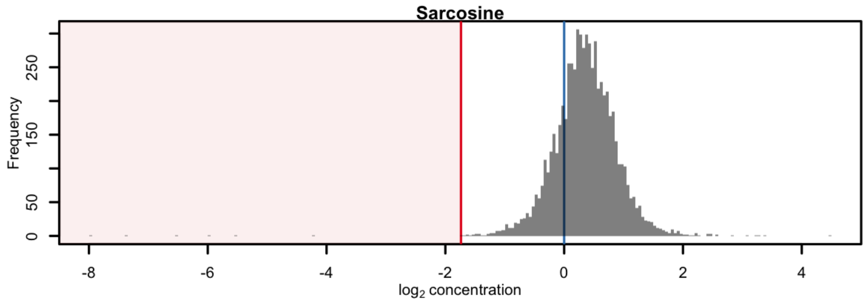

Figure S27: Signal distribution for Sarcosine.

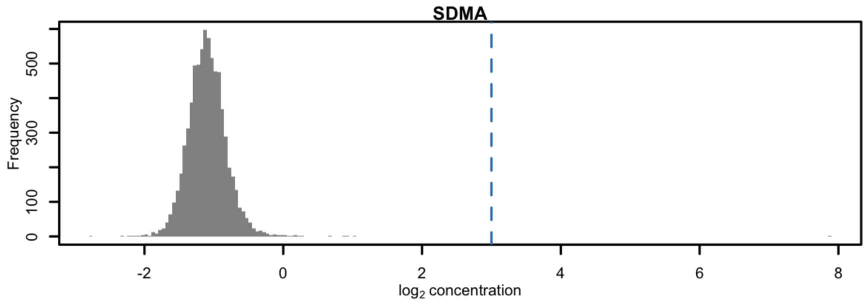

Figure S28: Signal distribution for SDMA.

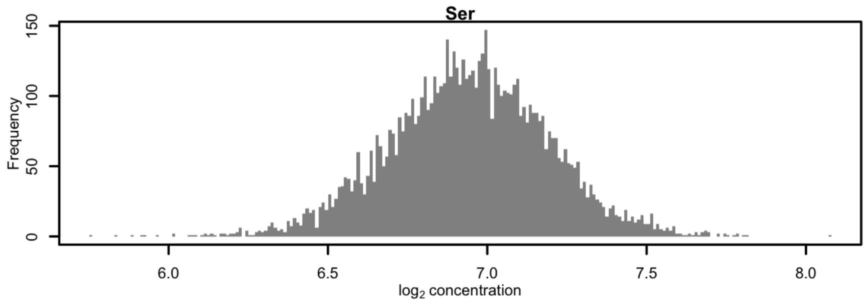

Figure S29: Signal distribution for Ser.

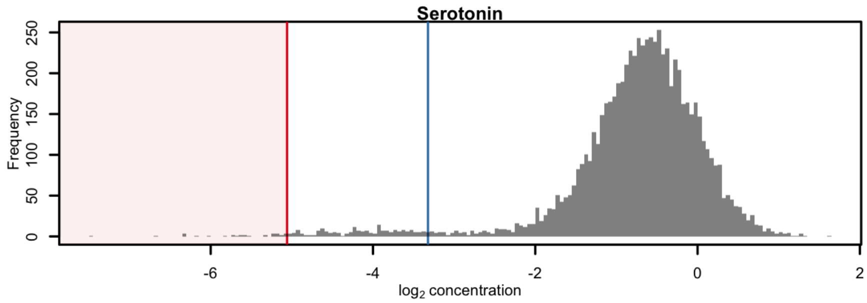

Figure S30: Signal distribution for Serotonin.

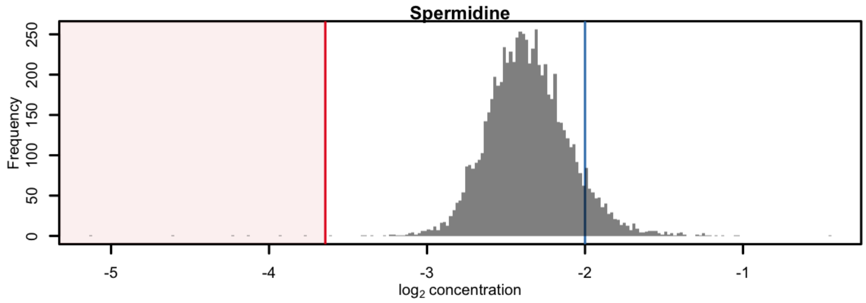

Figure S31: Signal distribution for Spermidine.

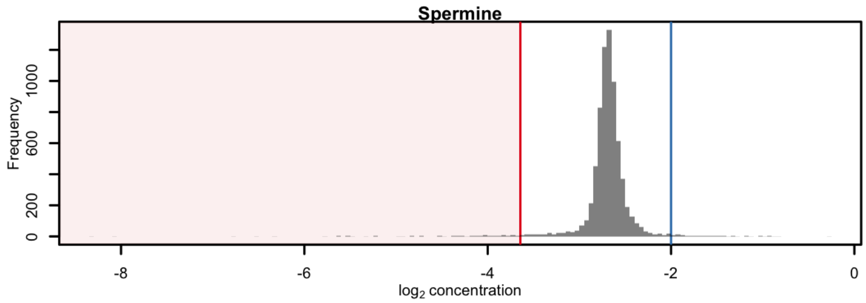

Figure S32: Signal distribution for Spermine.

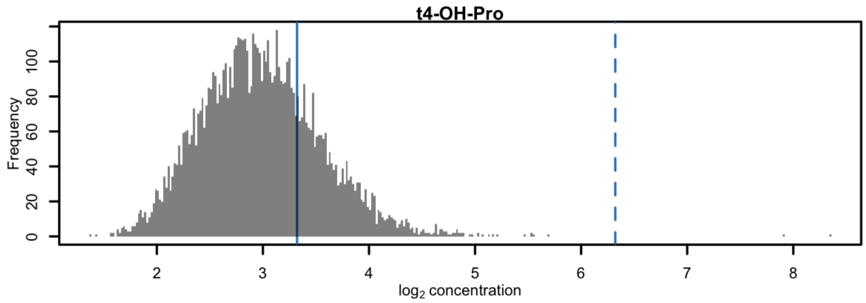

Figure S33: Signal distribution for t4-OH-Pro.

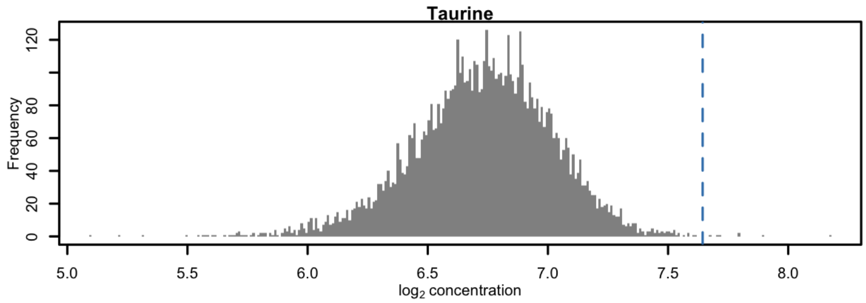

Figure S34: Signal distribution for Taurine.

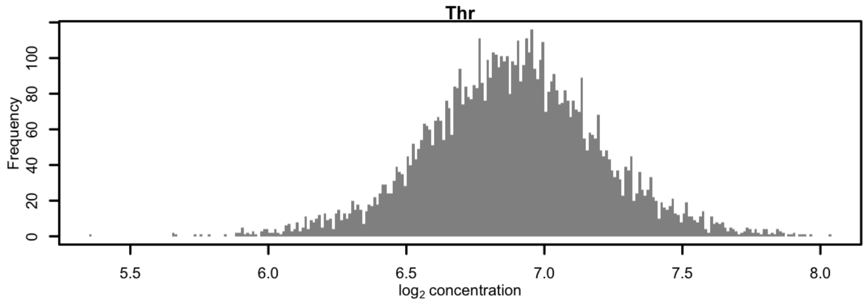

Figure S35: Signal distribution for Thr.

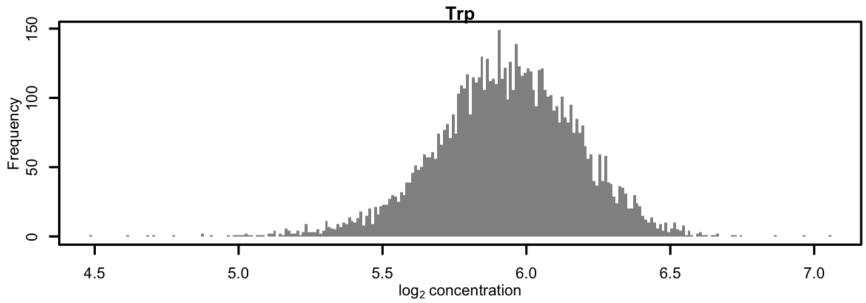

Figure S36: Signal distribution for Trp.

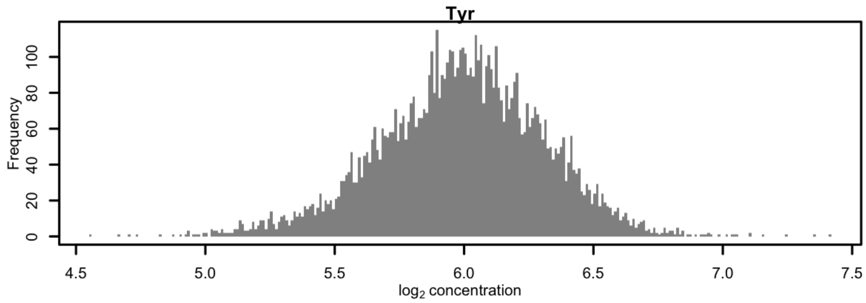

Figure S37: Signal distribution for Tyr.

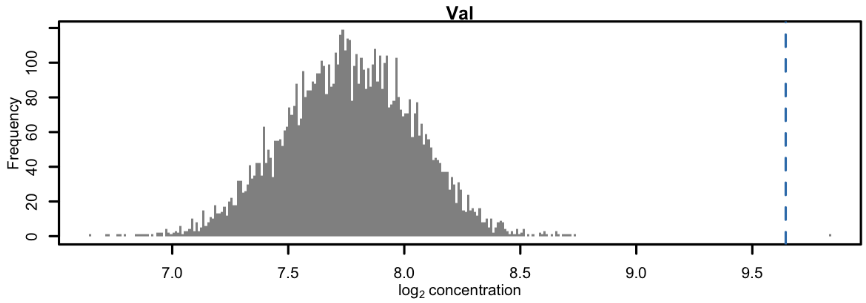

Figure S38: Signal distribution for Val.

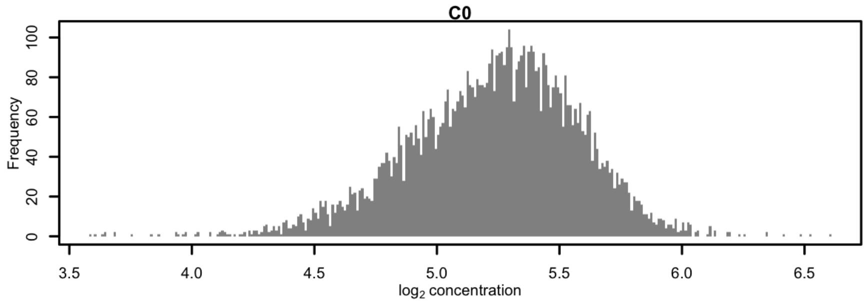

Figure S39: Signal distribution for C0.

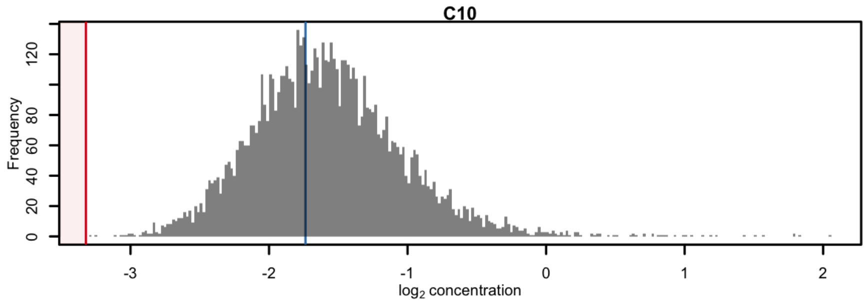

Figure S40: Signal distribution for C10.

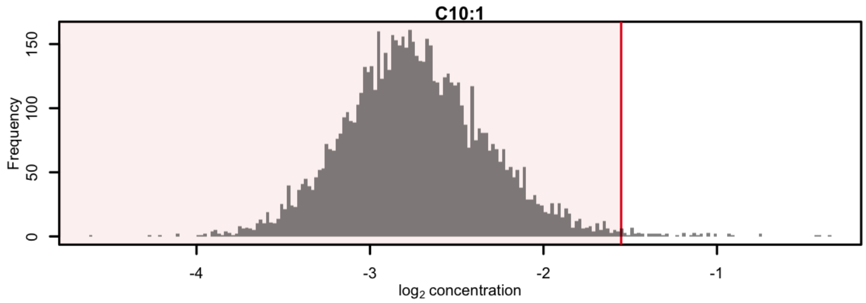

Figure S41: Signal distribution for C10:1.

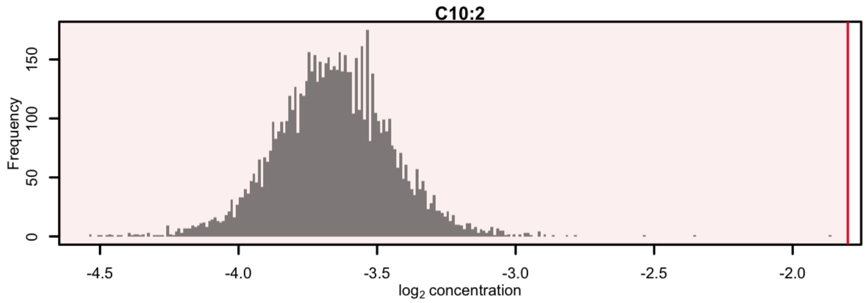

Figure S42: Signal distribution for C10:2.

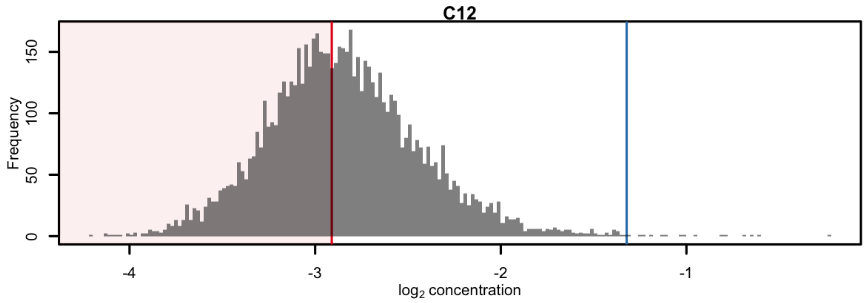

Figure S43: Signal distribution for C12.

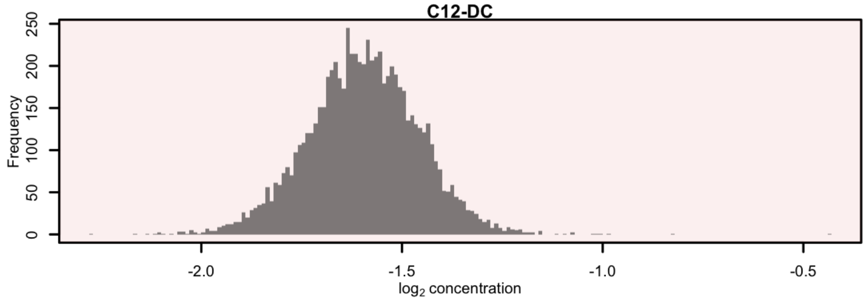

Figure S44: Signal distribution for C12-DC.

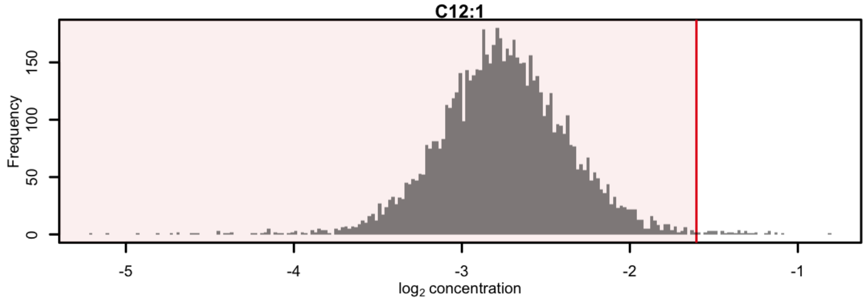

Figure S45: Signal distribution for C12:1.

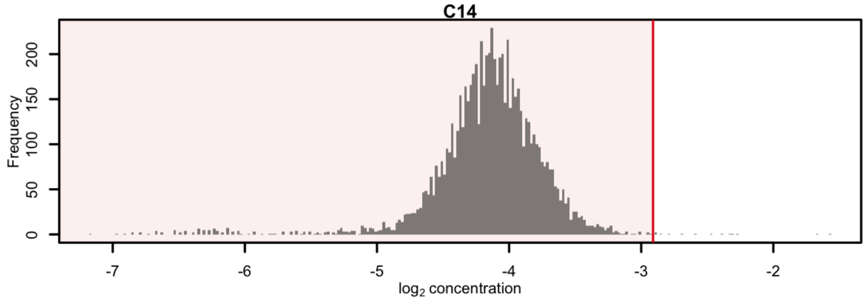

Figure S46: Signal distribution for C14.

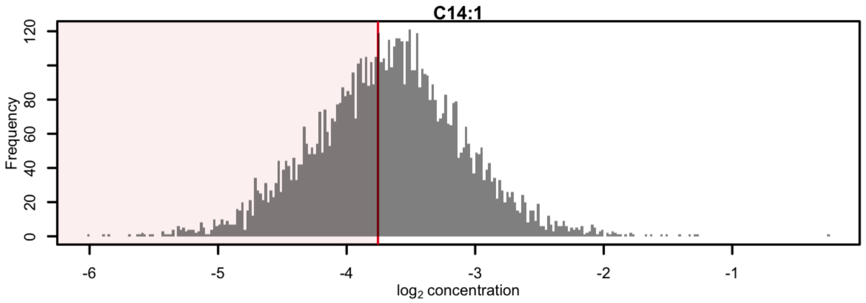

Figure S47: Signal distribution for C14:1.

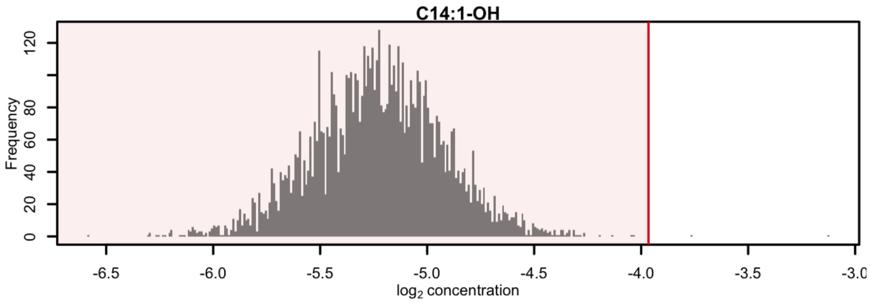

Figure S48: Signal distribution for C14:1-OH.

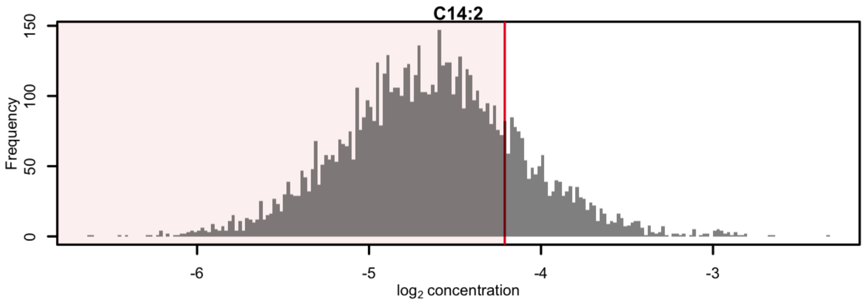

Figure S49: Signal distribution for C14:2.

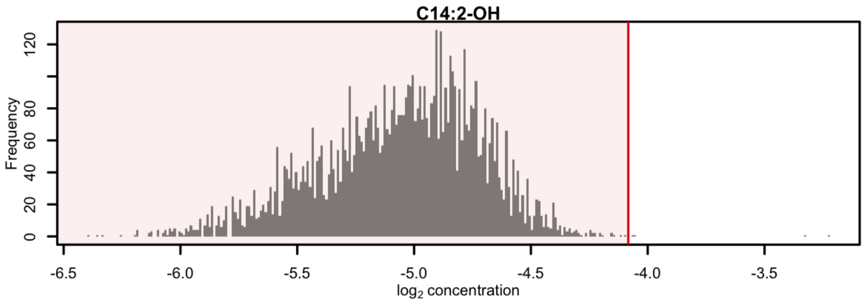

Figure S50: Signal distribution for C14:2-OH.

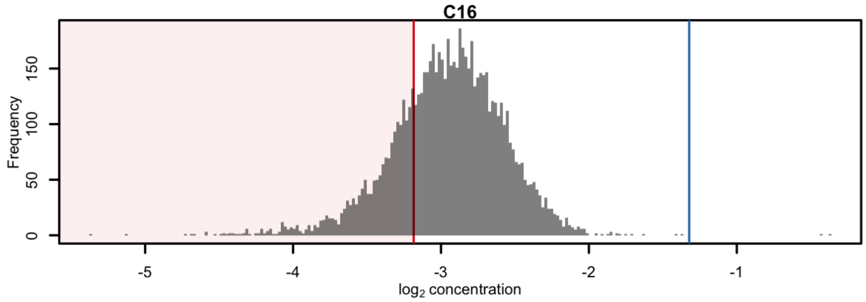

Figure S51: Signal distribution for C16.

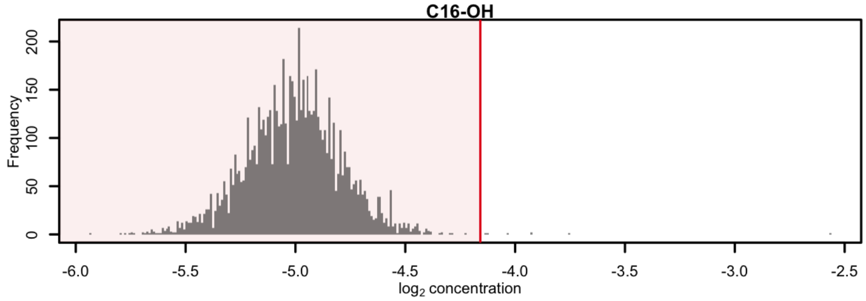

Figure S52: Signal distribution for C16-OH.

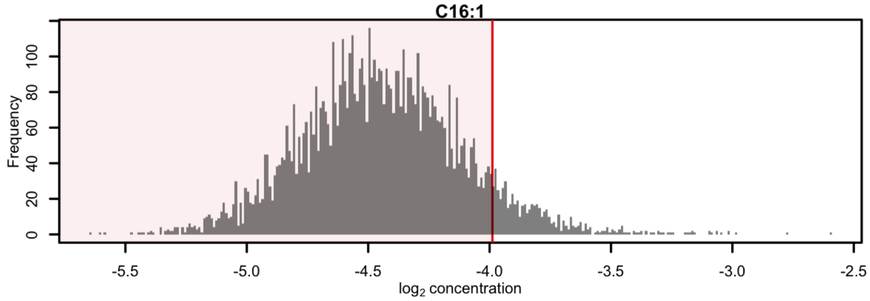

Figure S53: Signal distribution for C16:1.

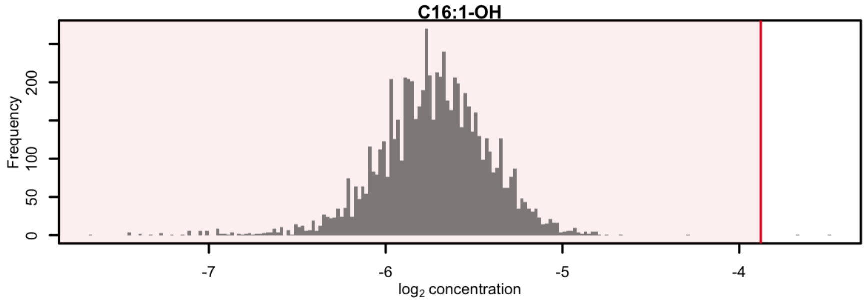

Figure S54: Signal distribution for C16:1-OH.

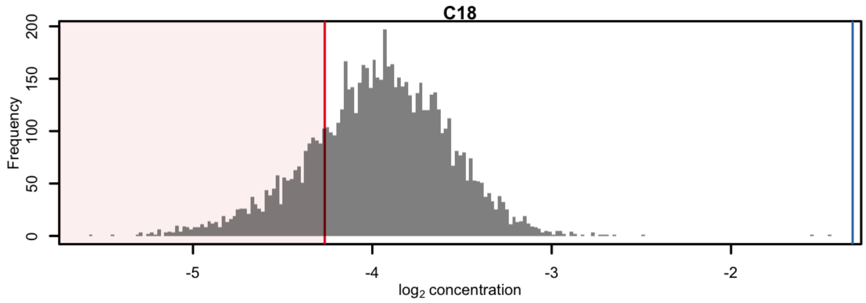

Figure S55: Signal distribution for C18.

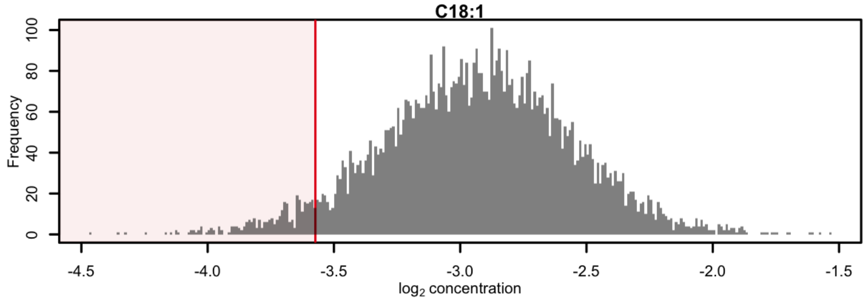

Figure S56: Signal distribution for C18:1.

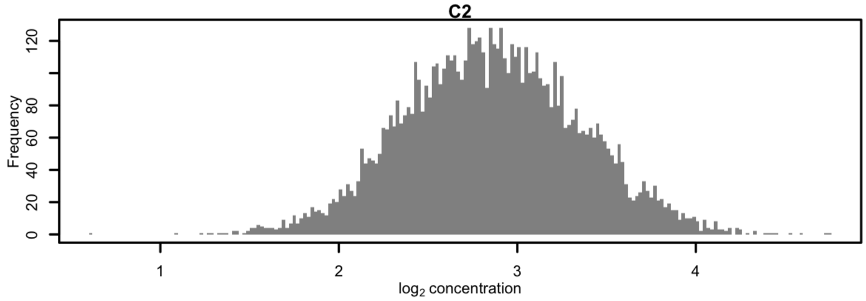

Figure S57: Signal distribution for C2.

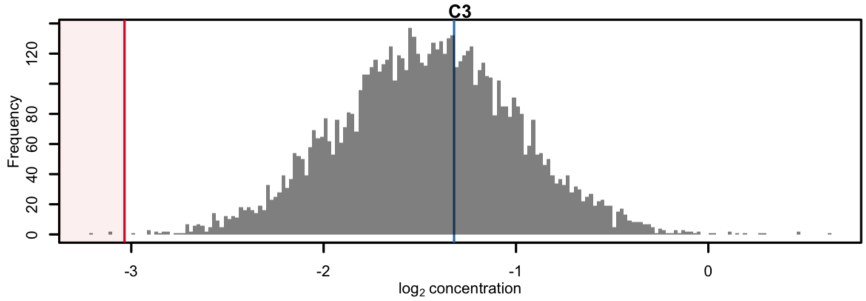

Figure S58: Signal distribution for C3.

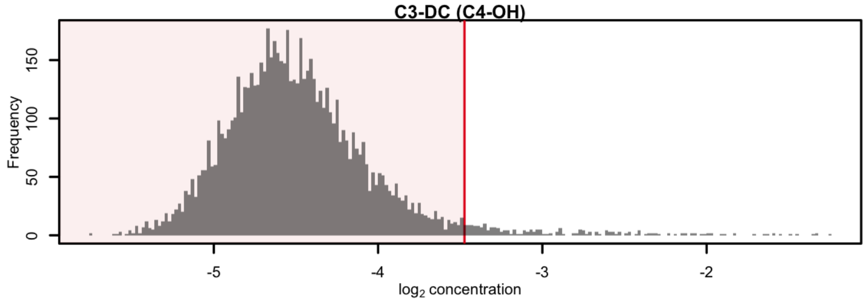

Figure S59: Signal distribution for C3-DC (C4-OH).

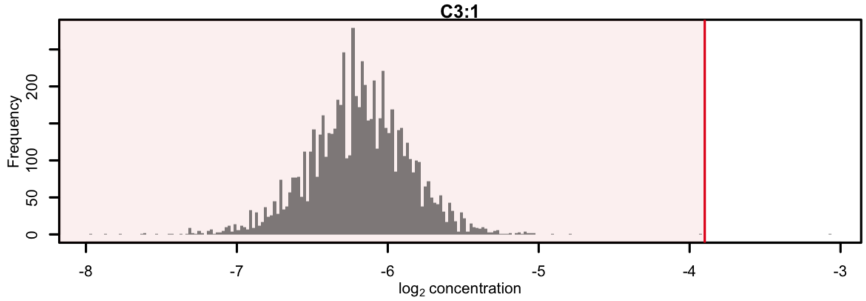

Figure S60: Signal distribution for C3:1.

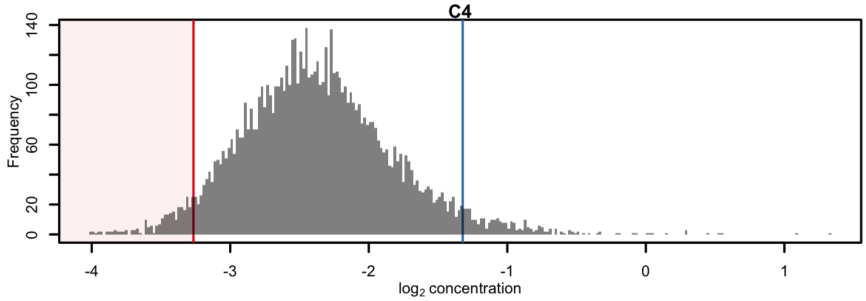

Figure S61: Signal distribution for C4.

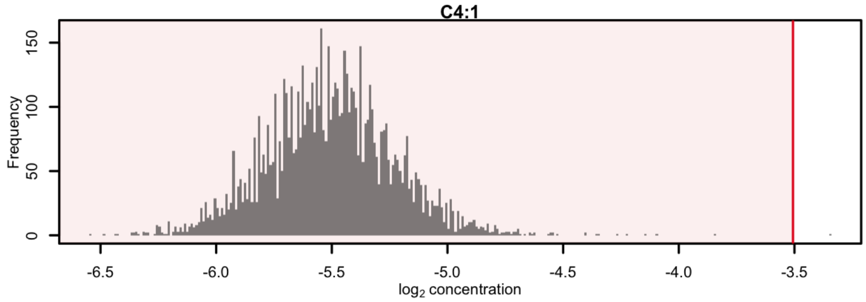

Figure S62: Signal distribution for C4:1.

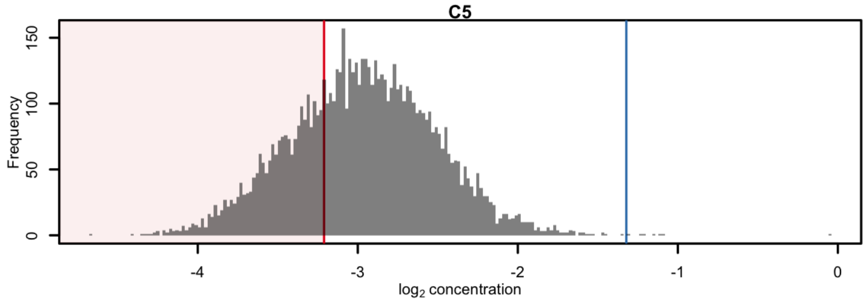

Figure S63: Signal distribution for C5.

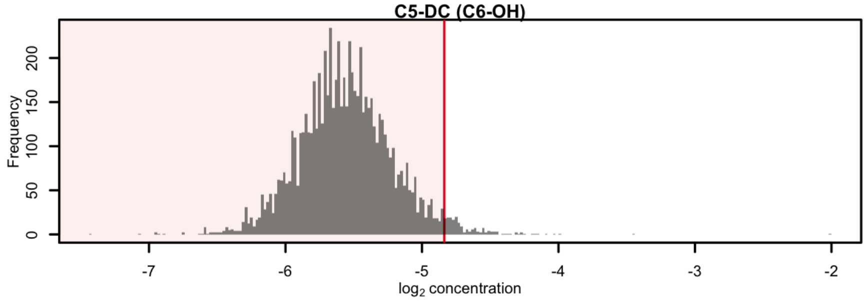

Figure S64: Signal distribution for C5-DC (C6-OH).

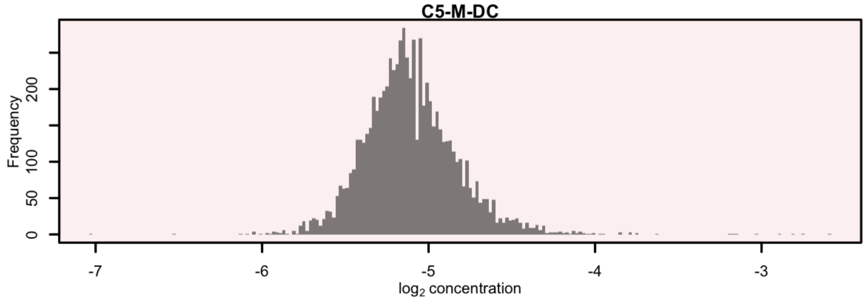

Figure S65: Signal distribution for C5-M-DC.

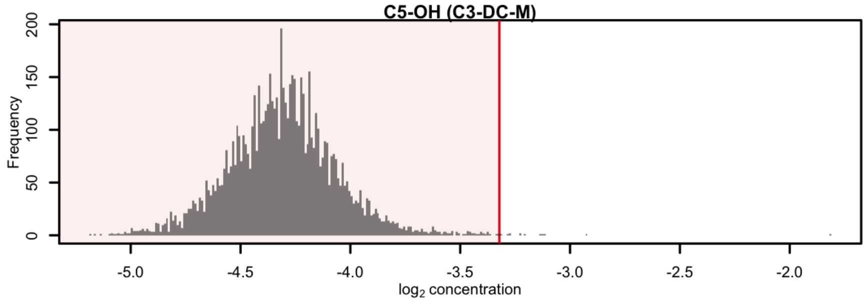

Figure S66: Signal distribution for C5-OH (C3-DC-M).

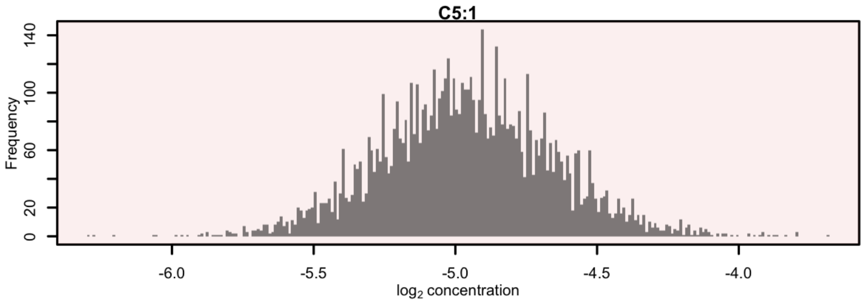

Figure S67: Signal distribution for C5:1.

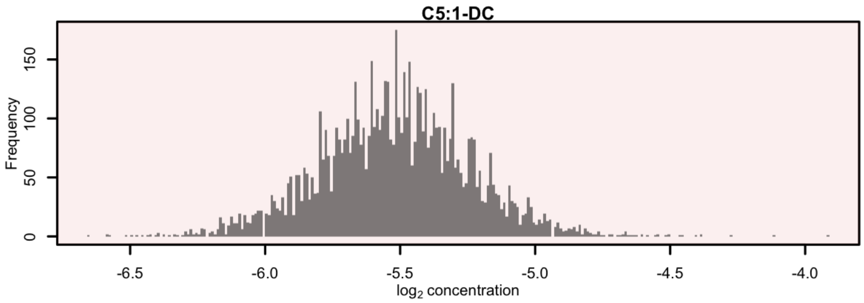

Figure S68: Signal distribution for C5:1-DC.

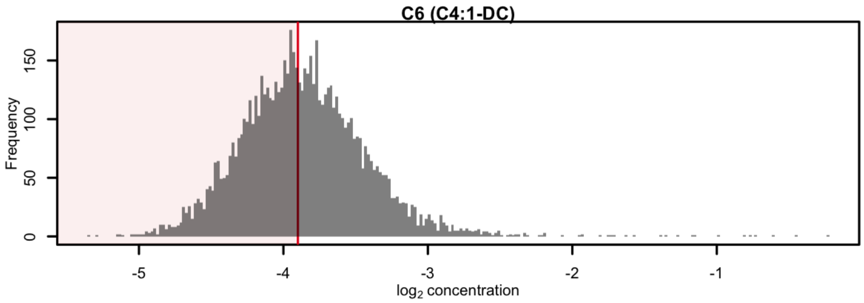

Figure S69: Signal distribution for C6 (C4:1-DC).

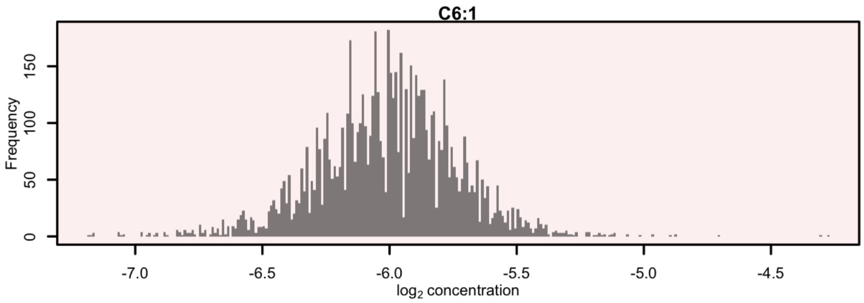

Figure S70: Signal distribution for C6:1.

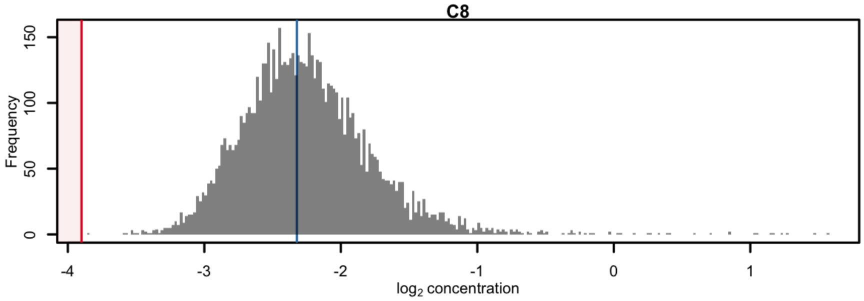

Figure S71: Signal distribution for C8.

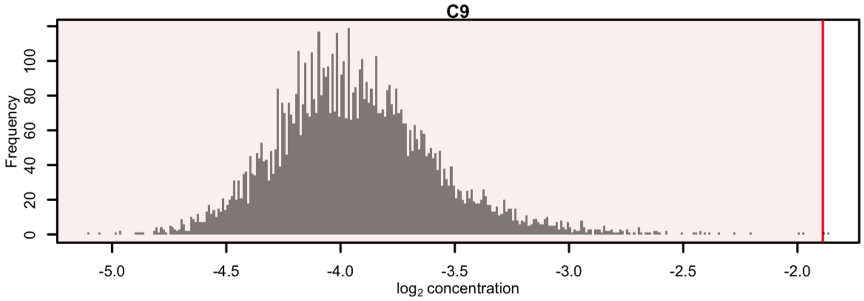

Figure S72: Signal distribution for C9.

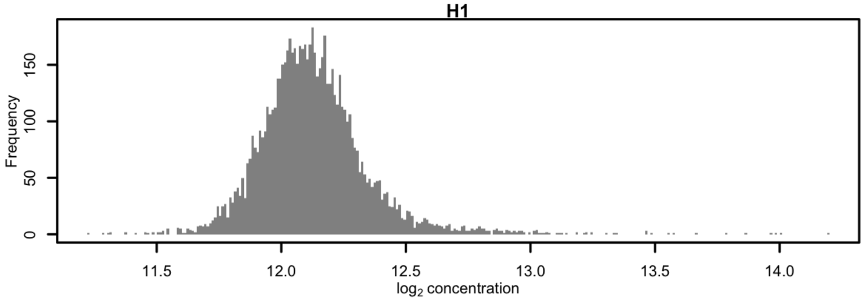

Figure S73: Signal distribution for H1.

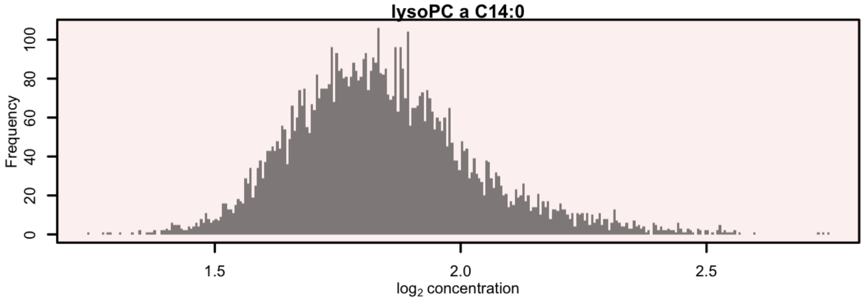

Figure S74: Signal distribution for lysoPC a C14:0.

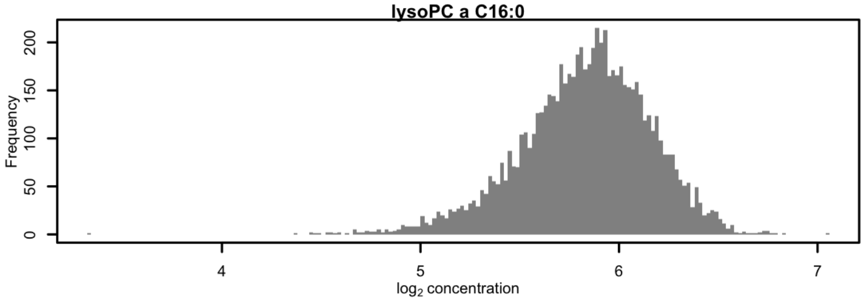

Figure S75: Signal distribution for lysoPC a C16:0.

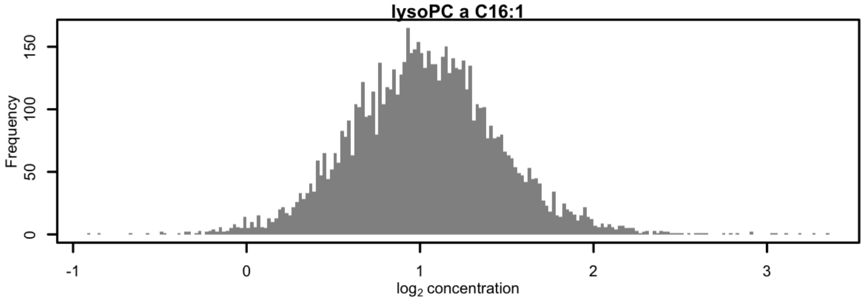

Figure S76: Signal distribution for lysoPC a C16:1.

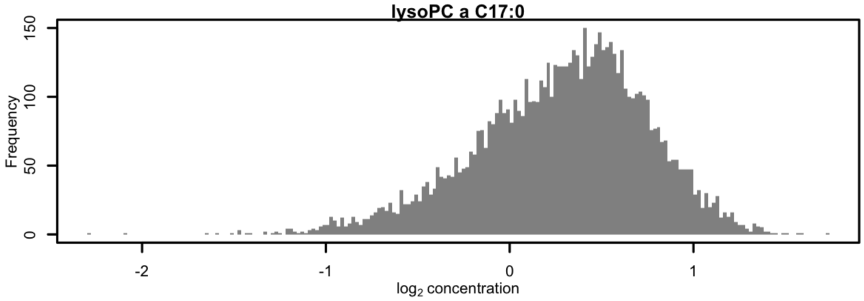

Figure S77: Signal distribution for lysoPC a C17:0.

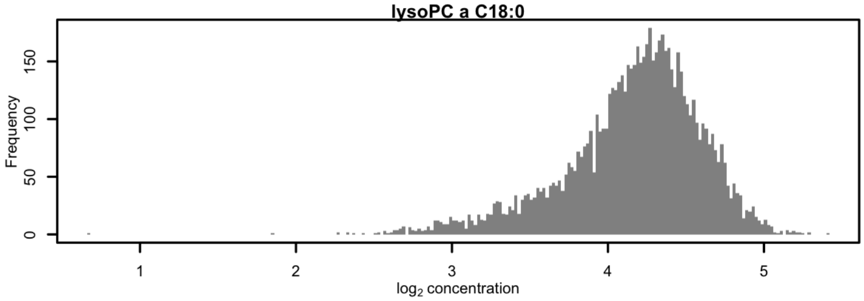

Figure S78: Signal distribution for lysoPC a C18:0.

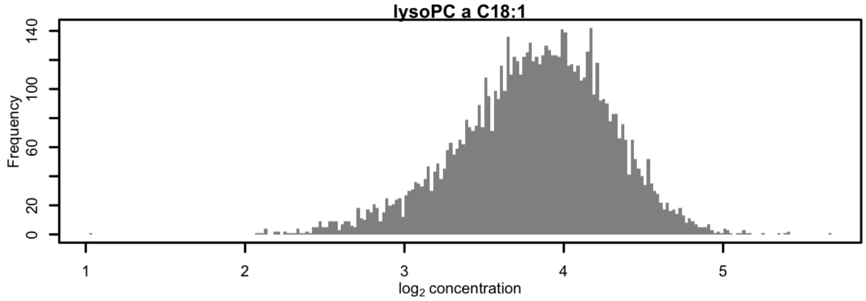

Figure S79: Signal distribution for lysoPC a C18:1.

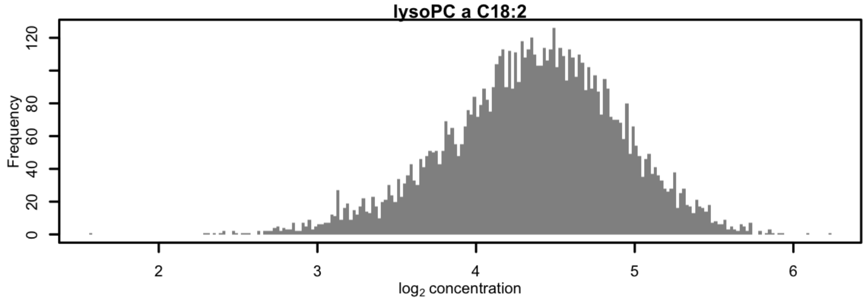

Figure S80: Signal distribution for lysoPC a C18:2.

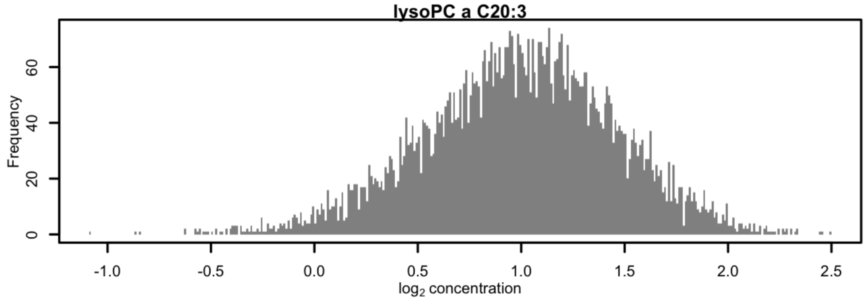

Figure S81: Signal distribution for lysoPC a C20:3.

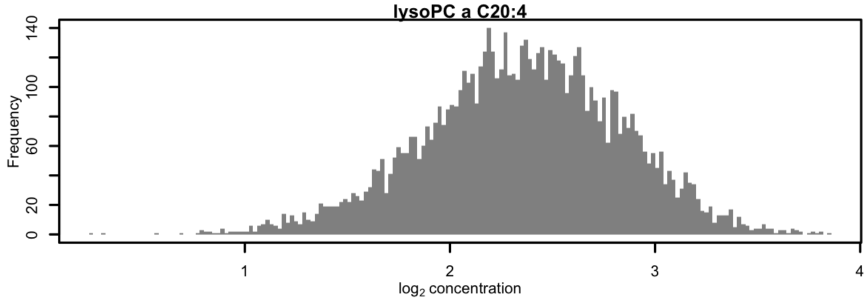

Figure S82: Signal distribution for lysoPC a C20:4.

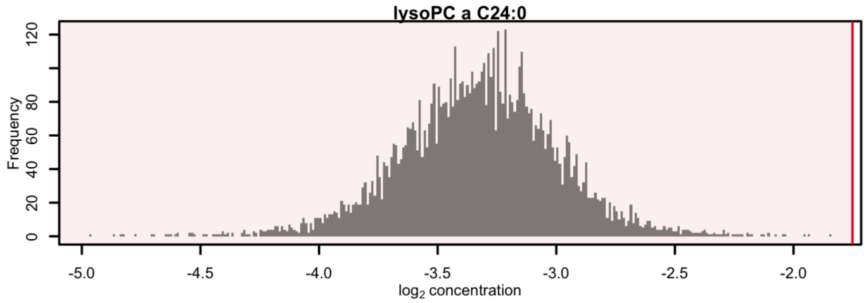

Figure S83: Signal distribution for lysoPC a C24:0.

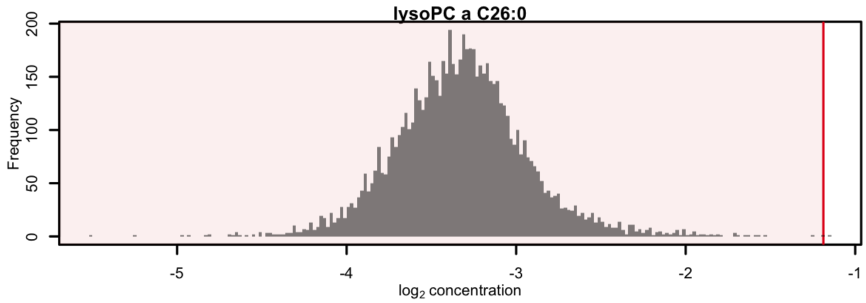

Figure S84: Signal distribution for lysoPC a C26:0.

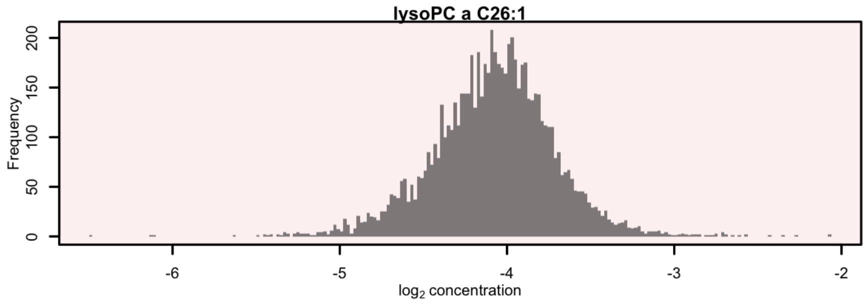

Figure S85: Signal distribution for lysoPC a C26:1.

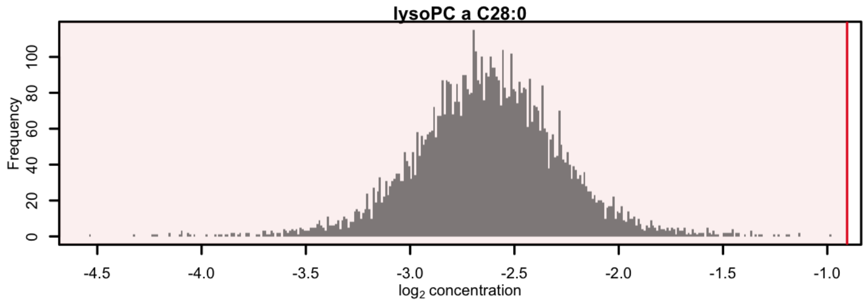

Figure S86: Signal distribution for lysoPC a C28:0.

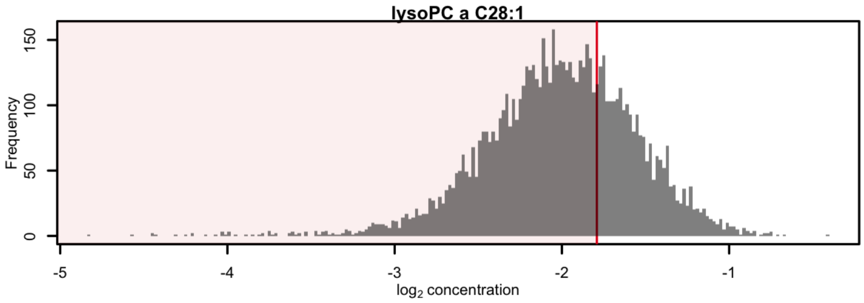

Figure S87: Signal distribution for lysoPC a C28:1.

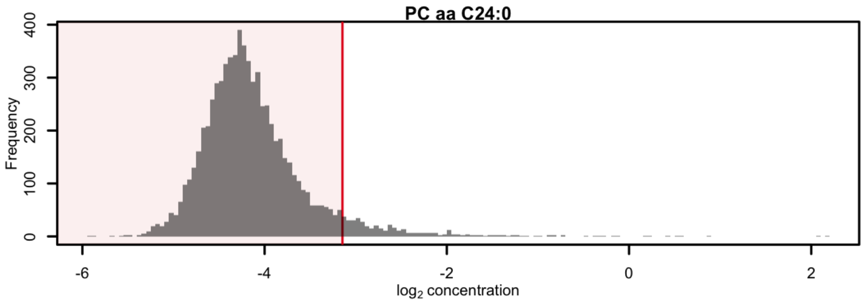

Figure S88: Signal distribution for PC aa C24:0.

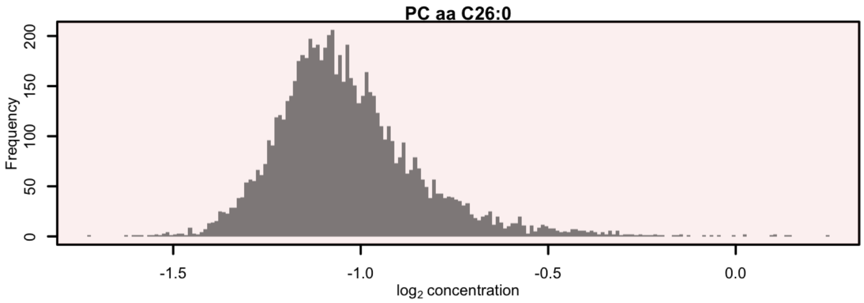

Figure S89: Signal distribution for PC aa C26:0.

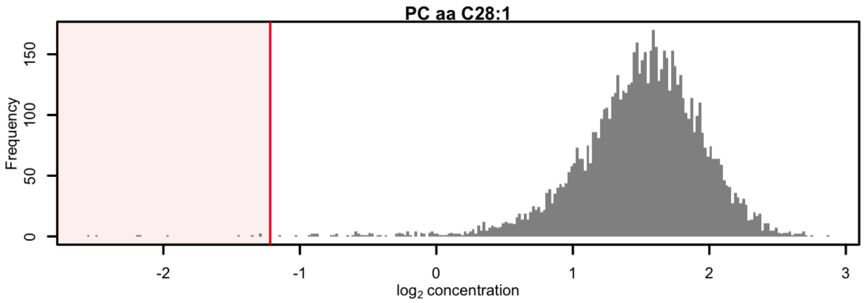

Figure S90: Signal distribution for PC aa C28:1.

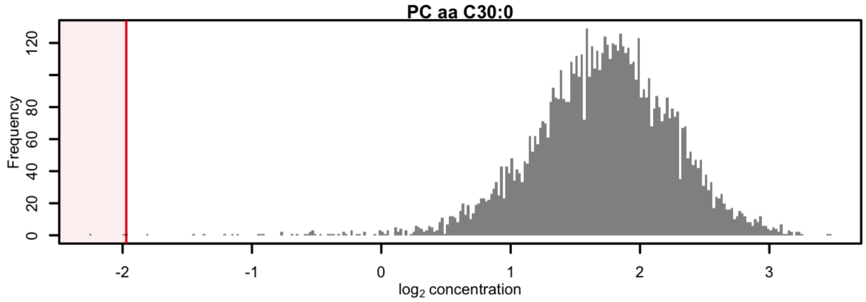

Figure S91: Signal distribution for PC aa C30:0.

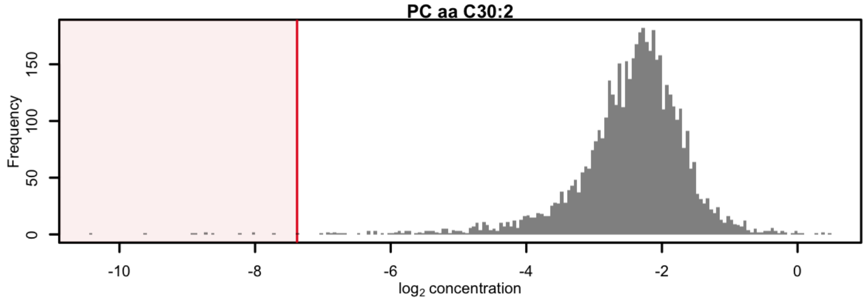

Figure S92: Signal distribution for PC aa C30:2.

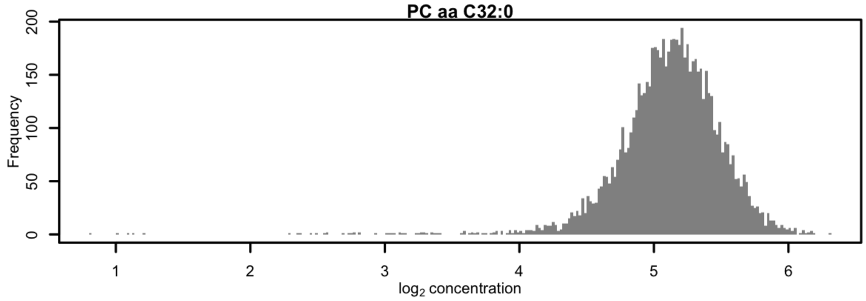

Figure S93: Signal distribution for PC aa C32:0.

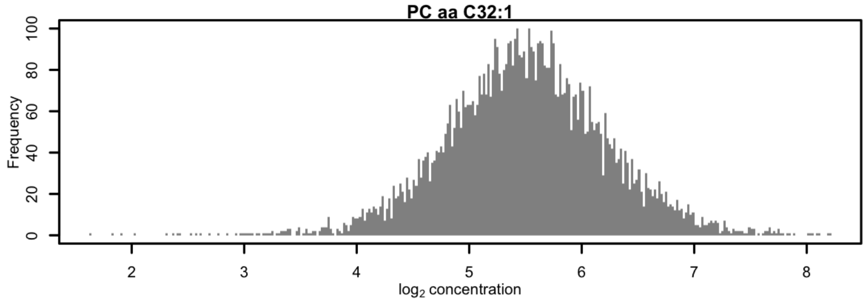

Figure S94: Signal distribution for PC aa C32:1.

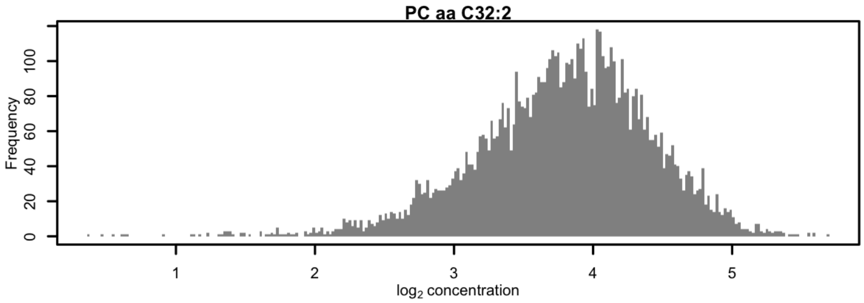

Figure S95: Signal distribution for PC aa C32:2.

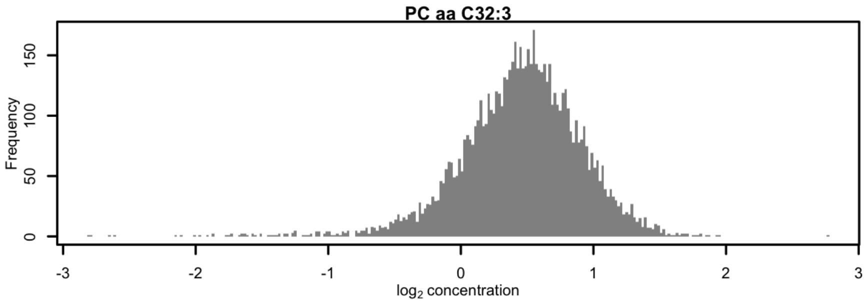

Figure S96: Signal distribution for PC aa C32:3.

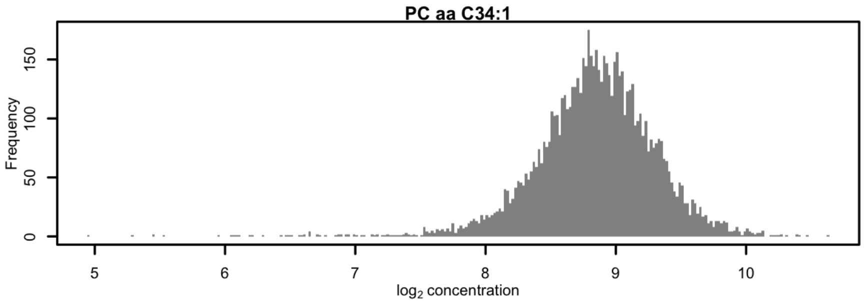

Figure S97: Signal distribution for PC aa C34:1.

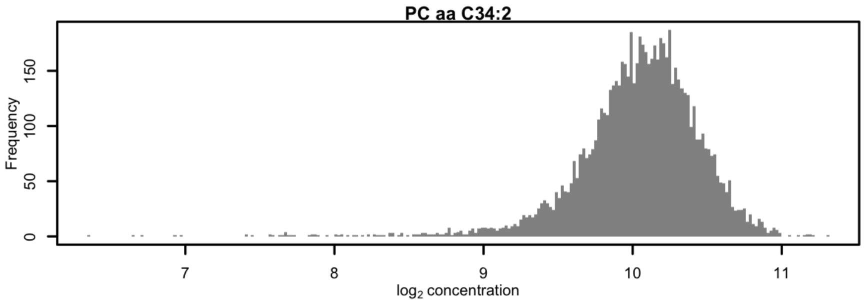

Figure S98: Signal distribution for PC aa C34:2.

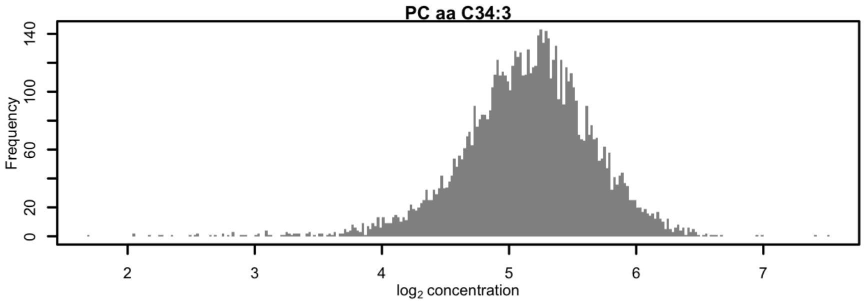

Figure S99: Signal distribution for PC aa C34:3.

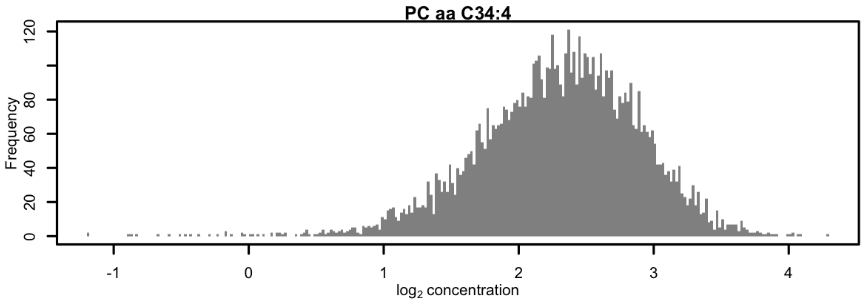

Figure S100: Signal distribution for PC aa C34:4.

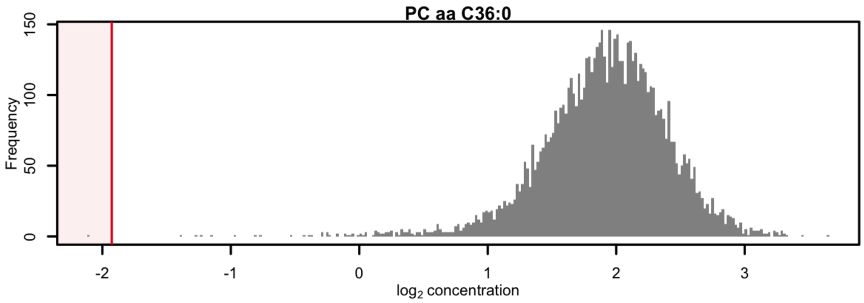

Figure S101: Signal distribution for PC aa C36:0.

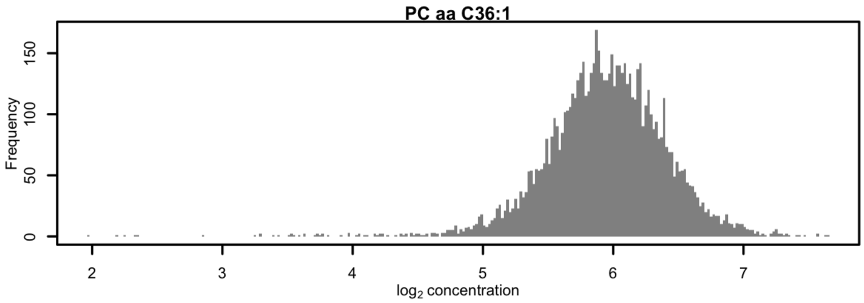

Figure S102: Signal distribution for PC aa C36:1.

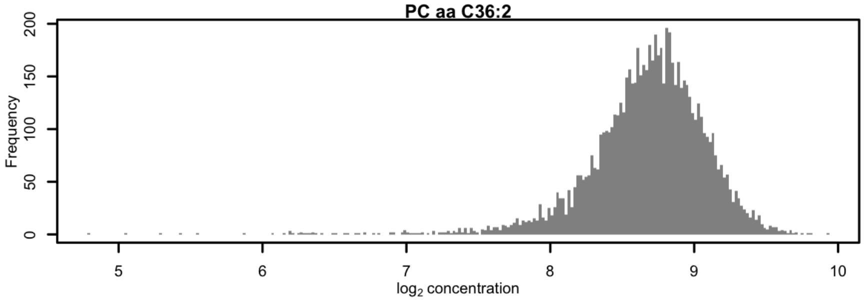

Figure S103: Signal distribution for PC aa C36:2.

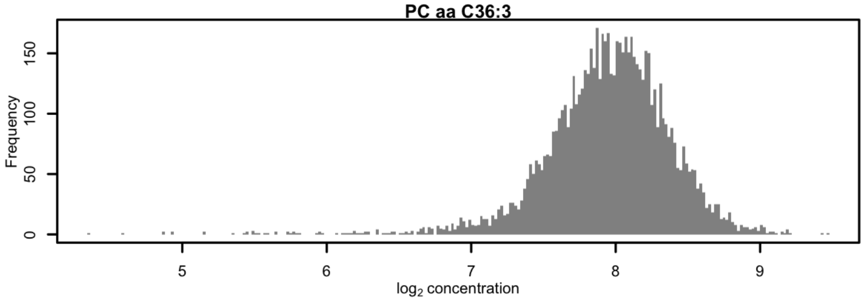

Figure S104: Signal distribution for PC aa C36:3.

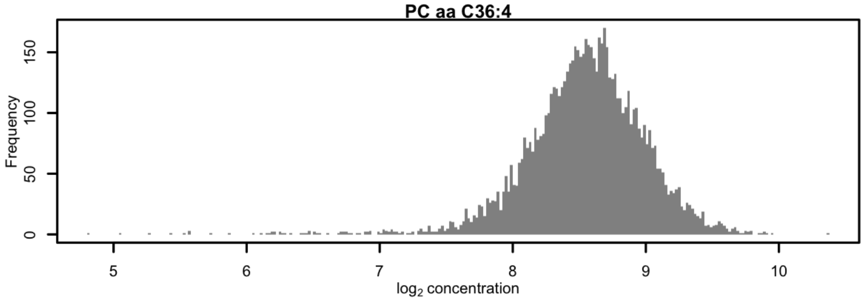

Figure S105: Signal distribution for PC aa C36:4.

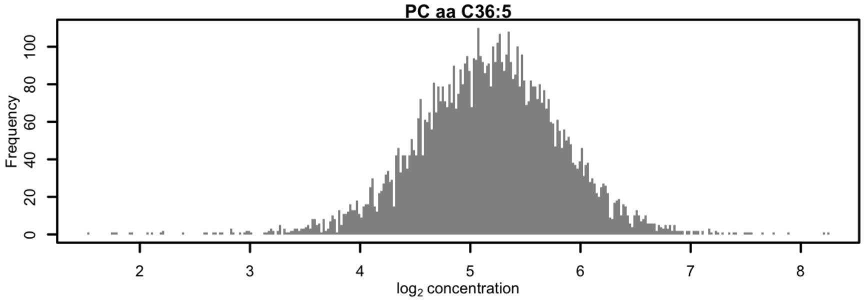

Figure S106: Signal distribution for PC aa C36:5.

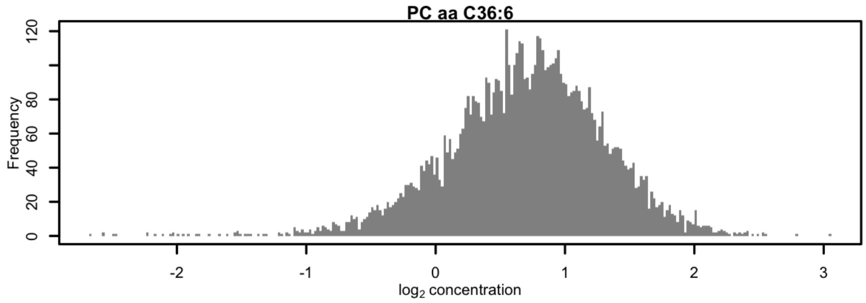

Figure S107: Signal distribution for PC aa C36:6.

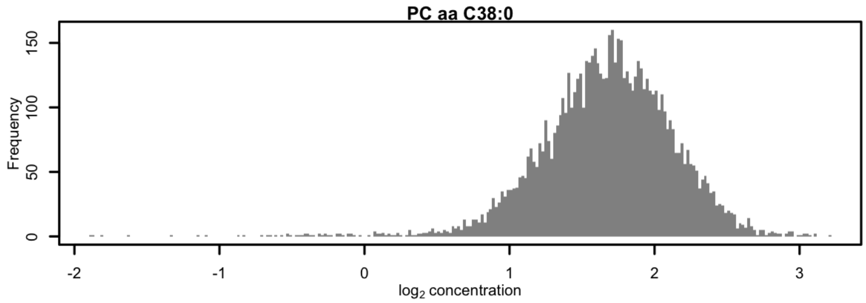

Figure S108: Signal distribution for PC aa C38:0.

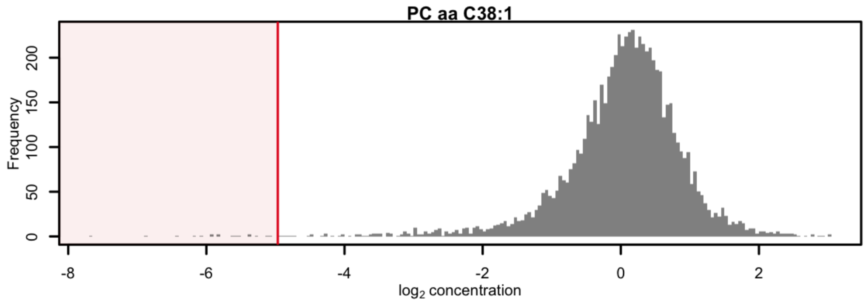

Figure S109: Signal distribution for PC aa C38:1.

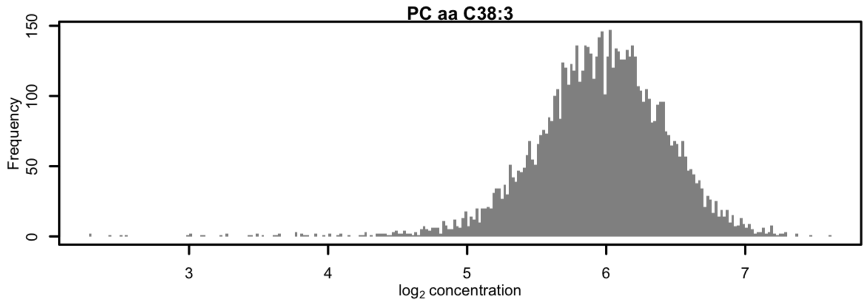

Figure S110: Signal distribution for PC aa C38:3.

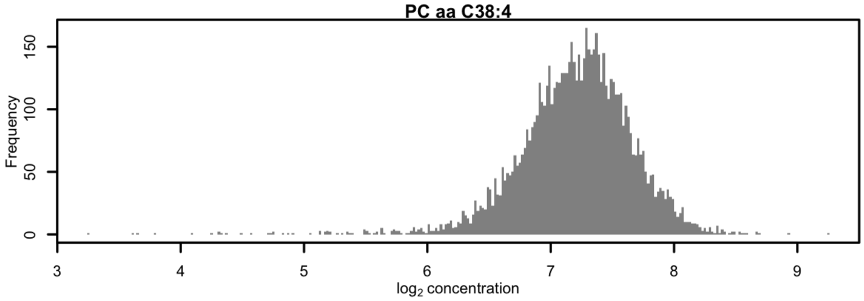

Figure S111: Signal distribution for PC aa C38:4.

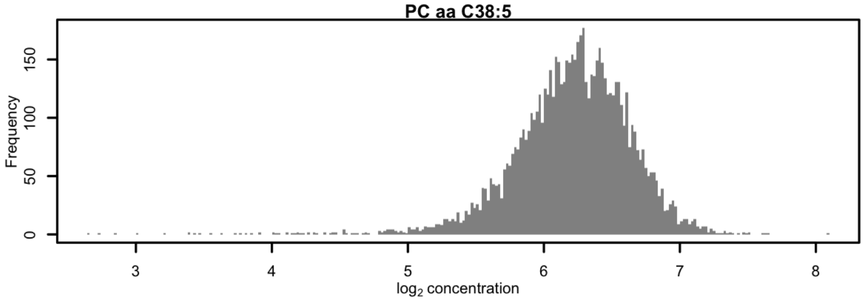

Figure S112: Signal distribution for PC aa C38:5.

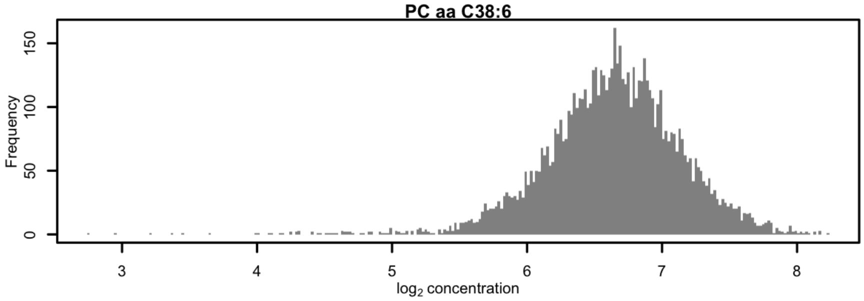

Figure S113: Signal distribution for PC aa C38:6.

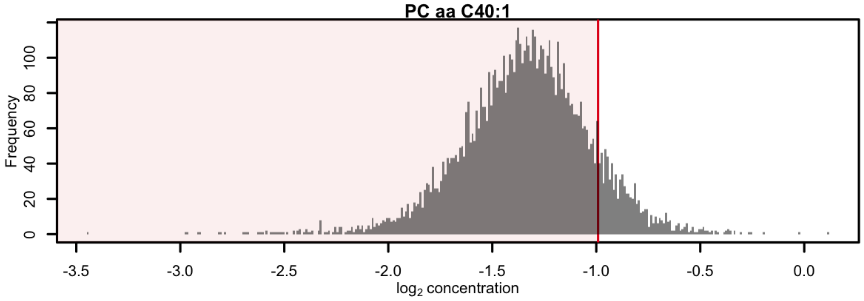

Figure S114: Signal distribution for PC aa C40:1.

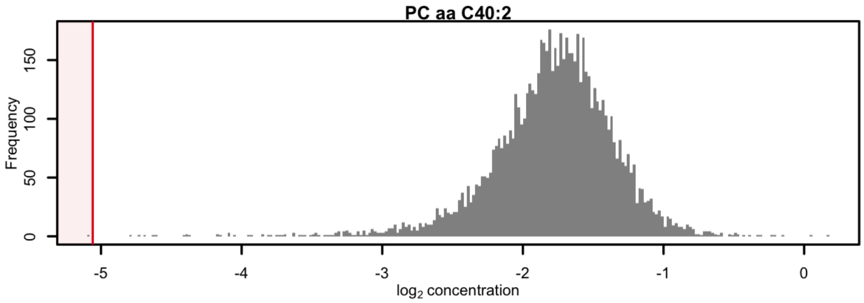

Figure S115: Signal distribution for PC aa C40:2.

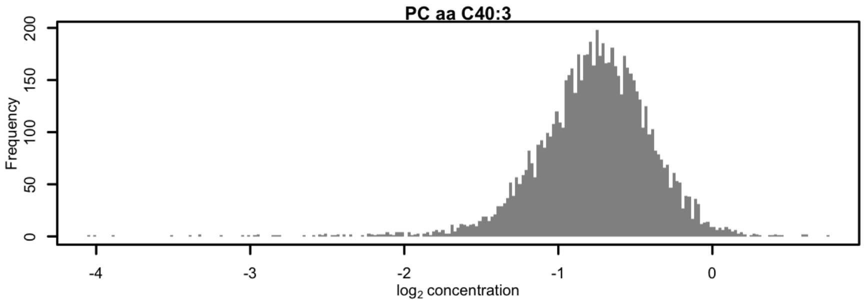

Figure S116: Signal distribution for PC aa C40:3.

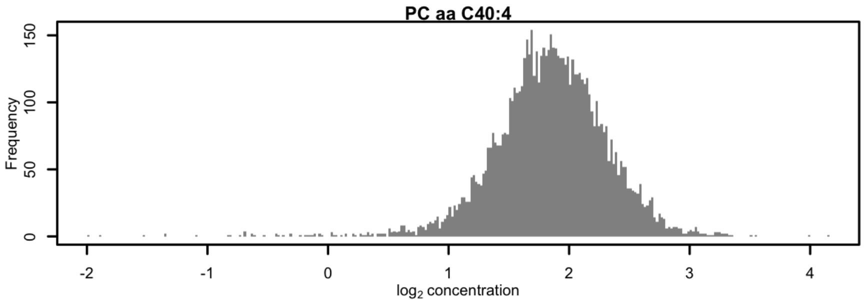

Figure S117: Signal distribution for PC aa C40:4.

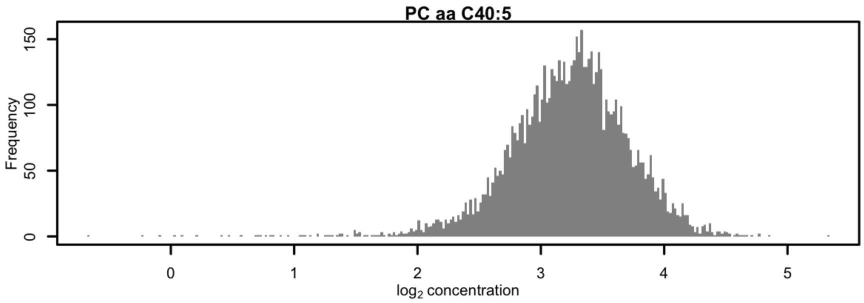

Figure S118: Signal distribution for PC aa C40:5.

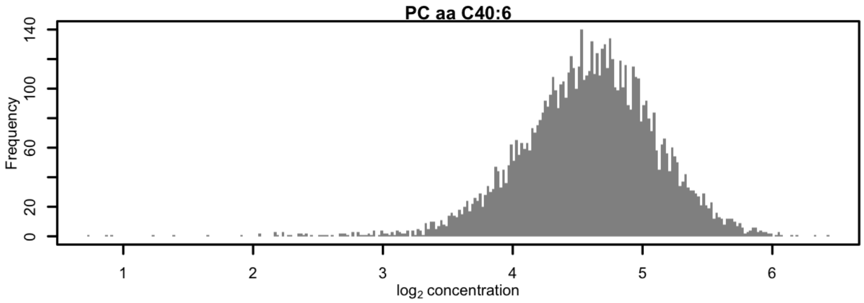

Figure S119: Signal distribution for PC aa C40:6.

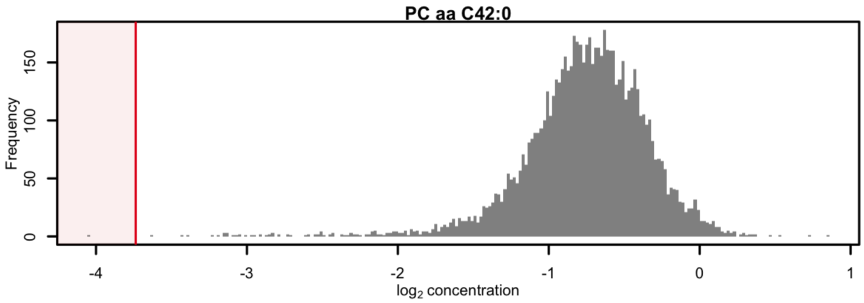

Figure S120: Signal distribution for PC aa C42:0.

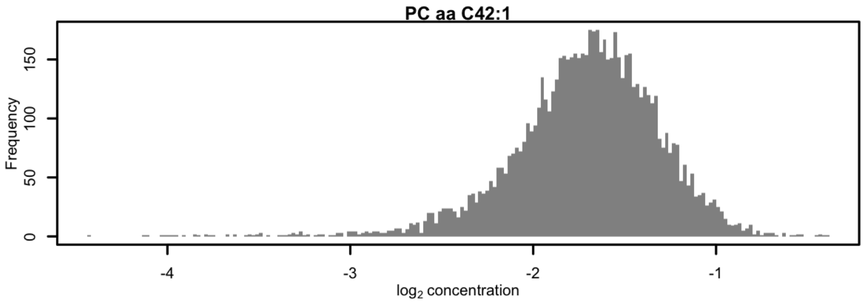

Figure S121: Signal distribution for PC aa C42:1.

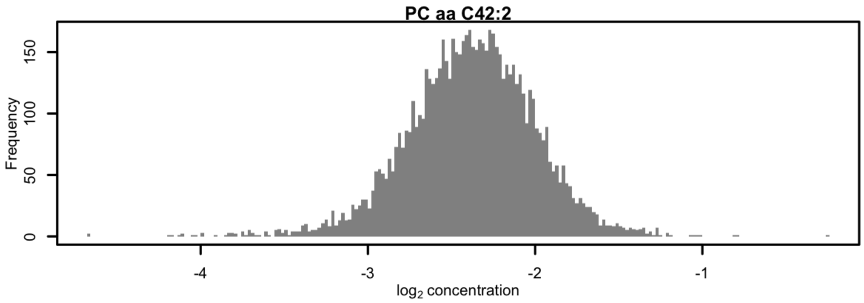

Figure S122: Signal distribution for PC aa C42:2.

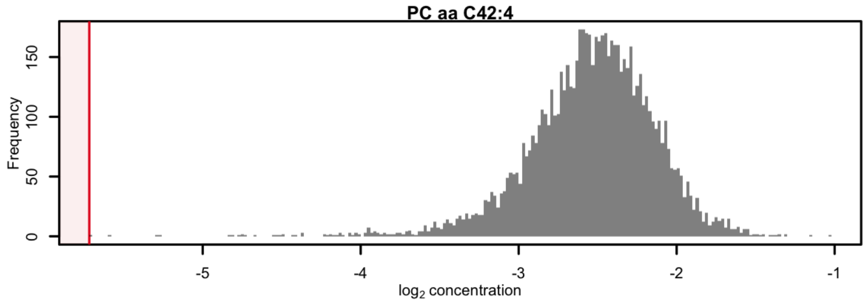

Figure S123: Signal distribution for PC aa C42:4.

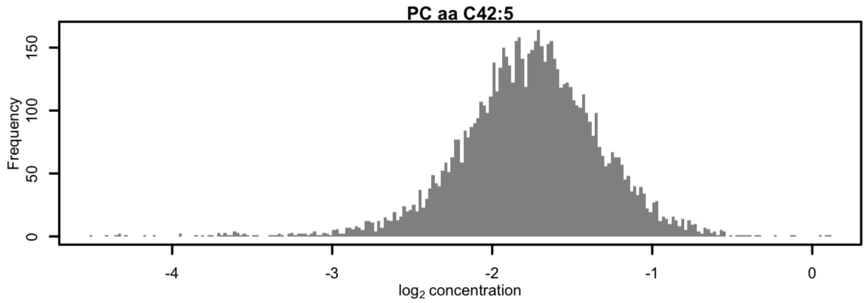

Figure S124: Signal distribution for PC aa C42:5.

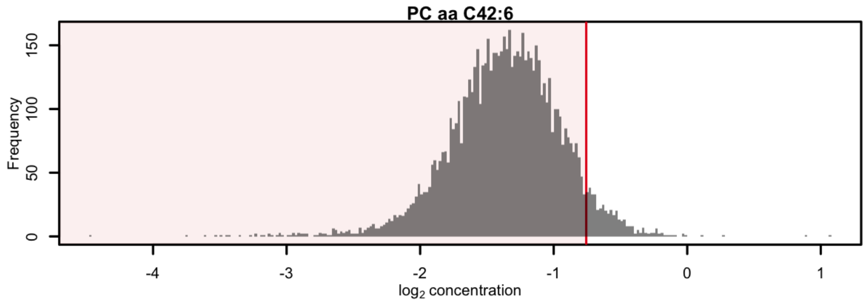

Figure S125: Signal distribution for PC aa C42:6.

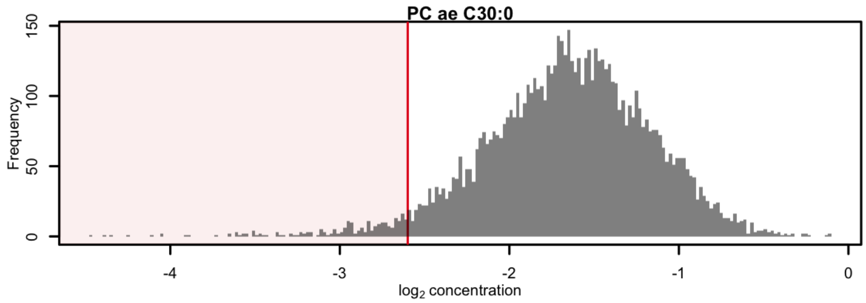

Figure S126: Signal distribution for PC ae C30:0.

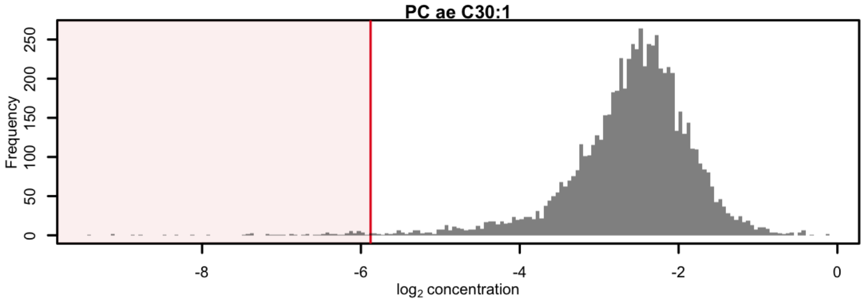

Figure S127: Signal distribution for PC ae C30:1.

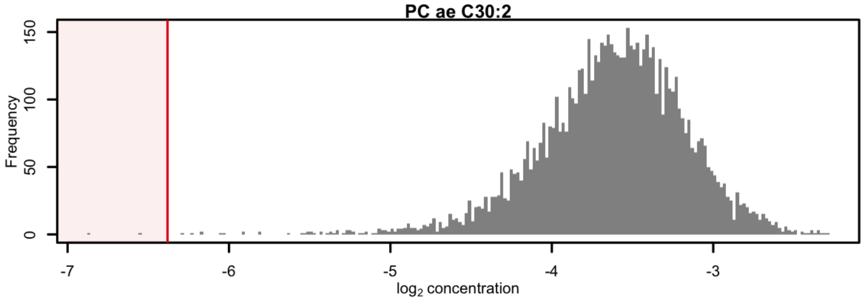

Figure S128: Signal distribution for PC ae C30:2.

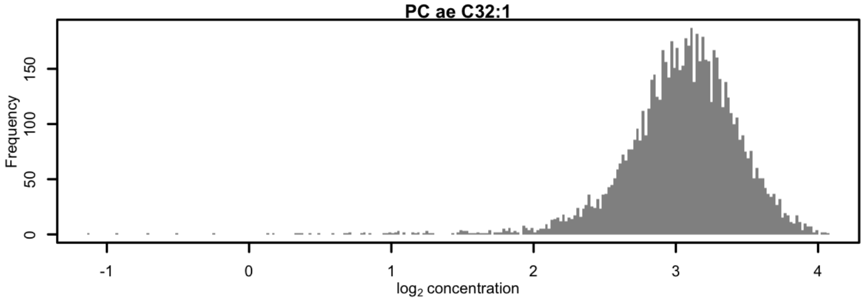

Figure S129: Signal distribution for PC ae C32:1.

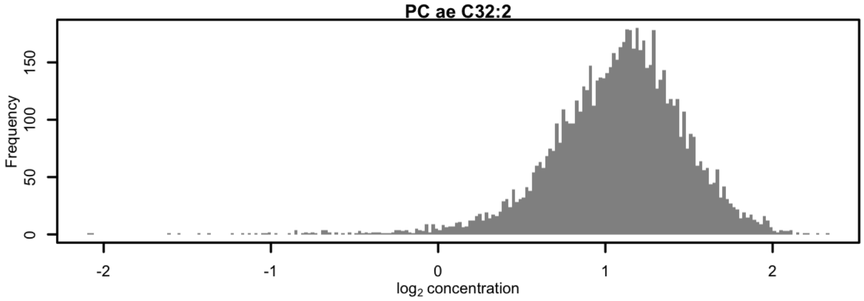

Figure S130: Signal distribution for PC ae C32:2.

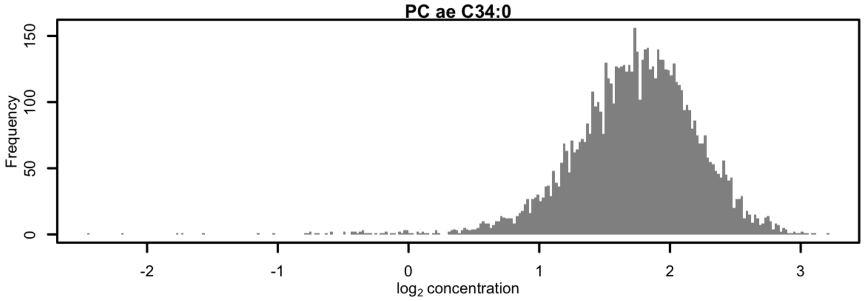

Figure S131: Signal distribution for PC ae C34:0.

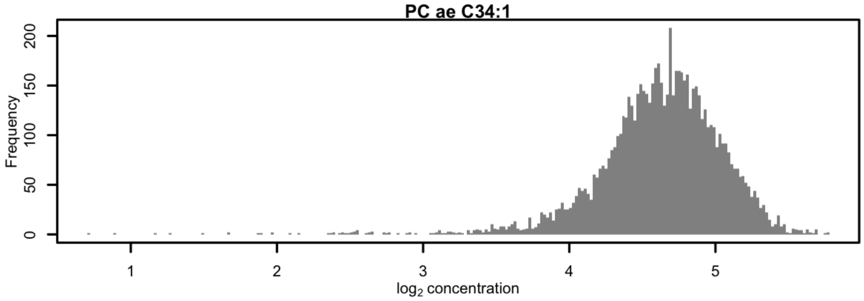

Figure S132: Signal distribution for PC ae C34:1.

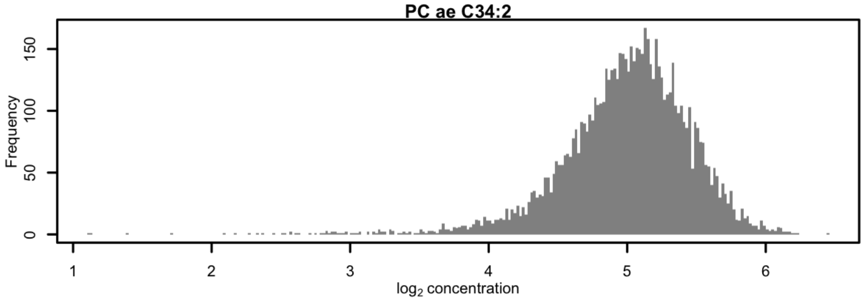

Figure S133: Signal distribution for PC ae C34:2.

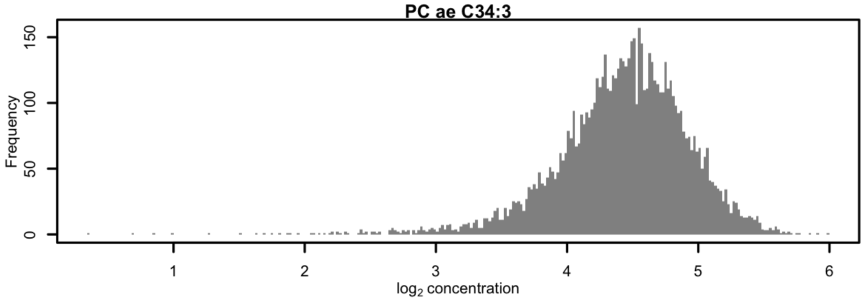

Figure S134: Signal distribution for PC ae C34:3.

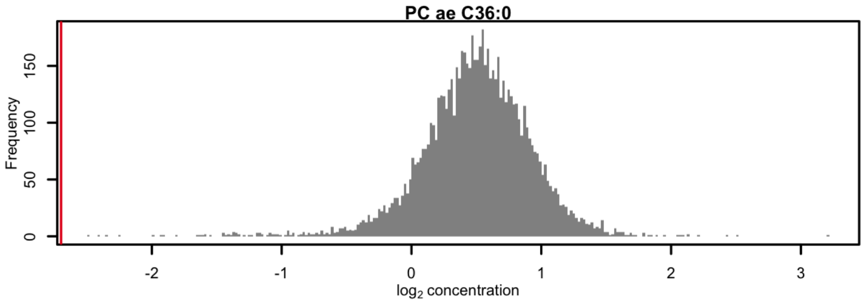

Figure S135: Signal distribution for PC ae C36:0.

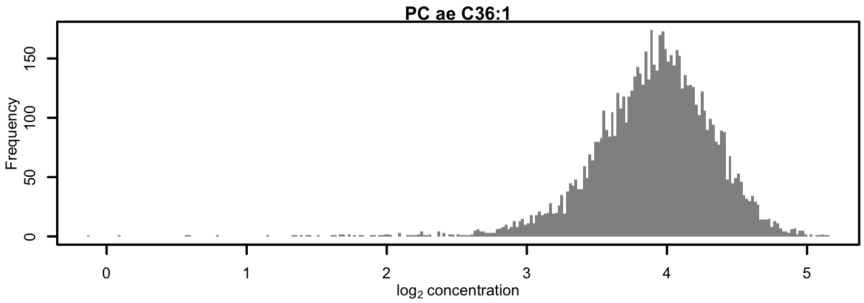

Figure S136: Signal distribution for PC ae C36:1.

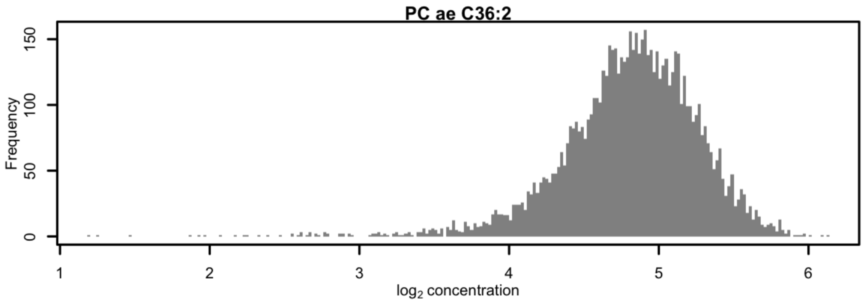

Figure S137: Signal distribution for PC ae C36:2.

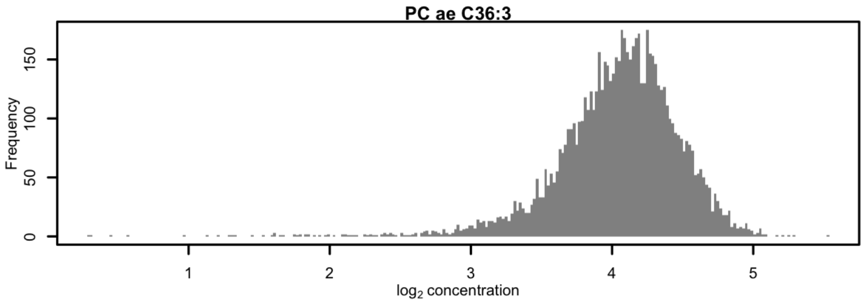

Figure S138: Signal distribution for PC ae C36:3.

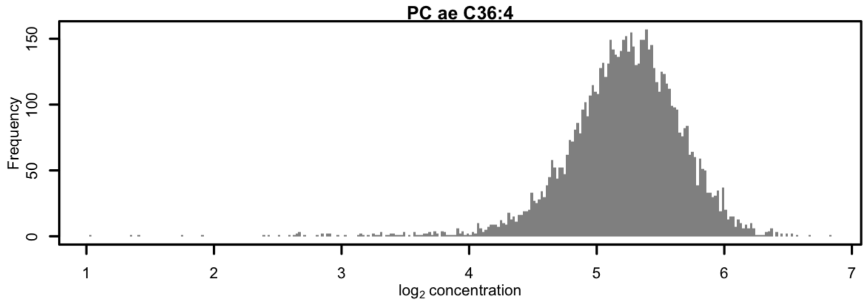

Figure S139: Signal distribution for PC ae C36:4.

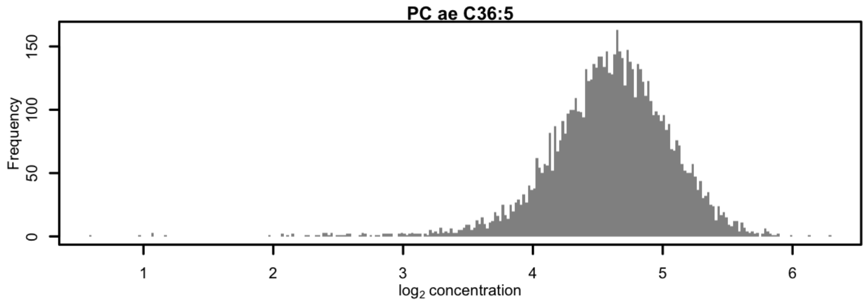

Figure S140: Signal distribution for PC ae C36:5.

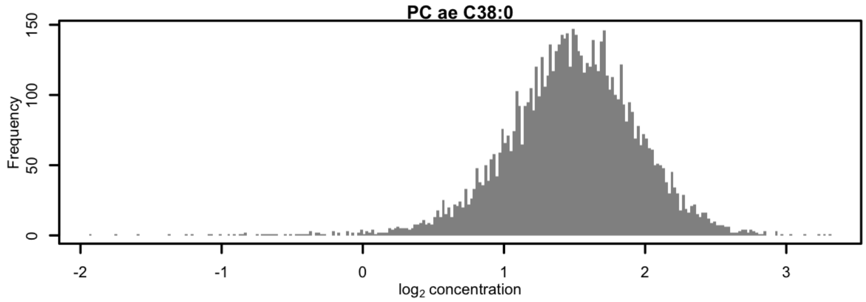

Figure S141: Signal distribution for PC ae C38:0.

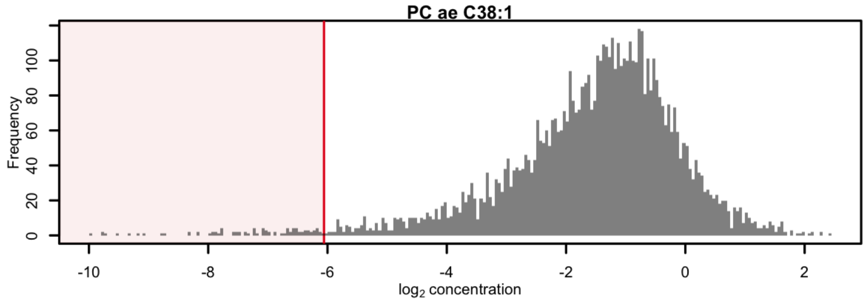

Figure S142: Signal distribution for PC ae C38:1.

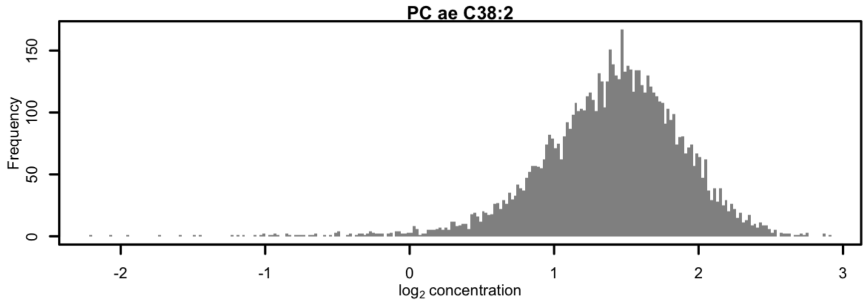

Figure S143: Signal distribution for PC ae C38:2.

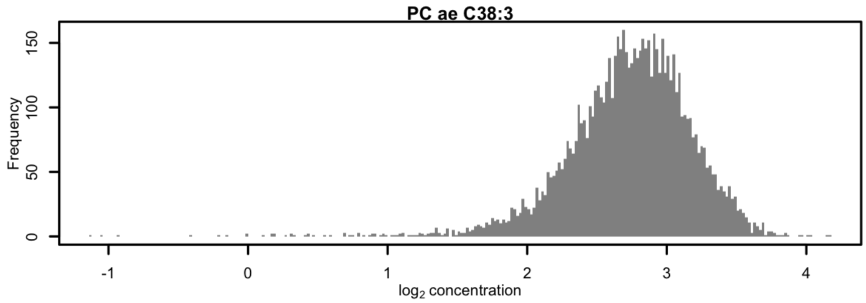

Figure S144: Signal distribution for PC ae C38:3.

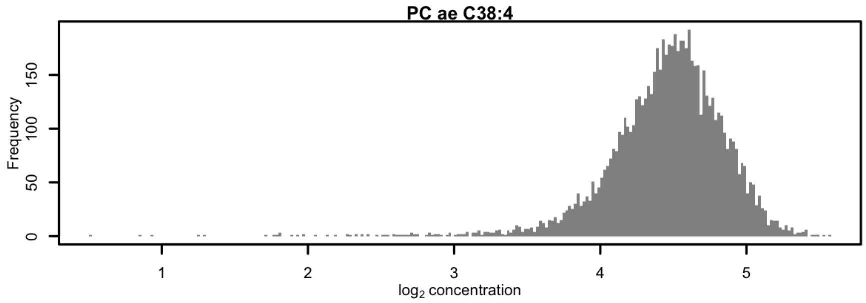

Figure S145: Signal distribution for PC ae C38:4.

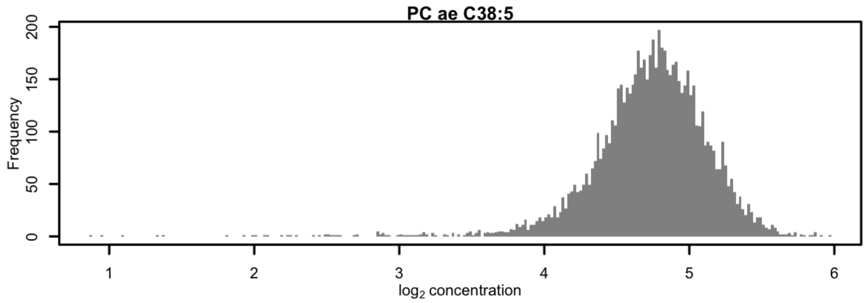

Figure S146: Signal distribution for PC ae C38:5.

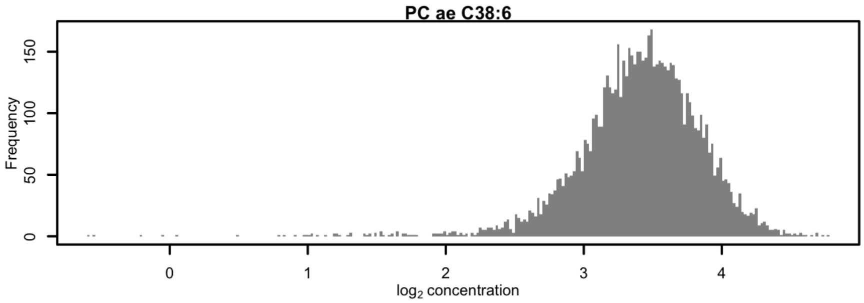

Figure S147: Signal distribution for PC ae C38:6.

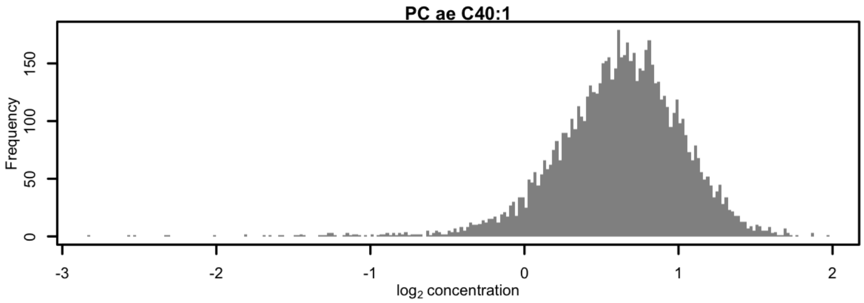

Figure S148: Signal distribution for PC ae C40:1.

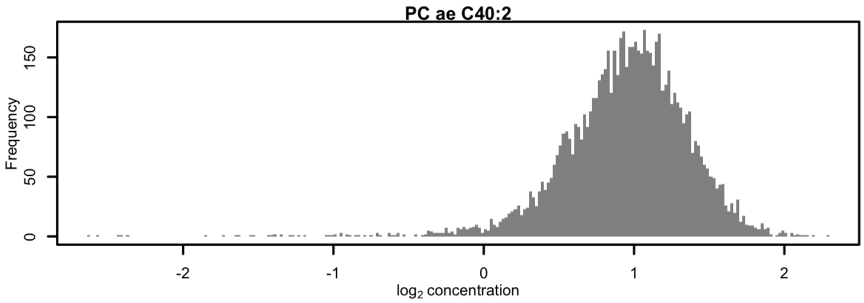

Figure S149: Signal distribution for PC ae C40:2.

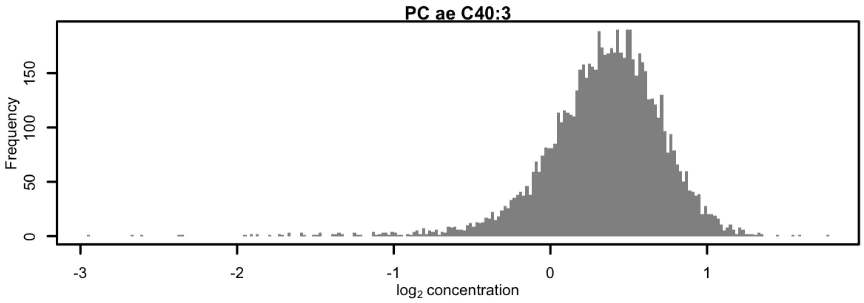

Figure S150: Signal distribution for PC ae C40:3.

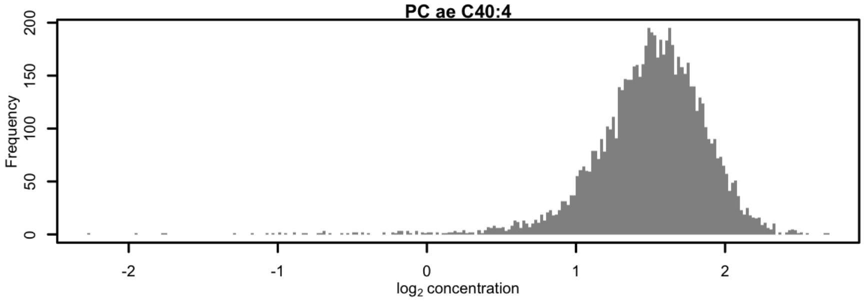

Figure S151: Signal distribution for PC ae C40:4.

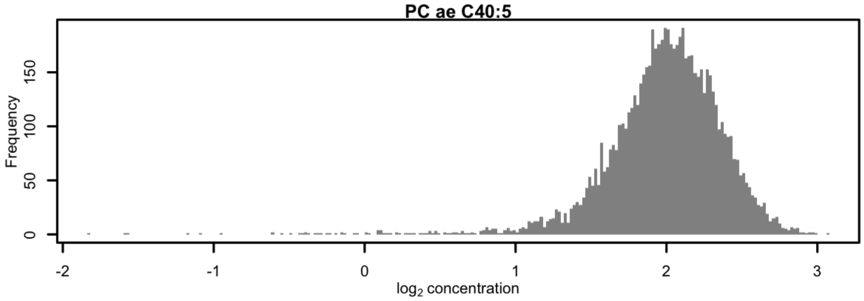

Figure S152: Signal distribution for PC ae C40:5.

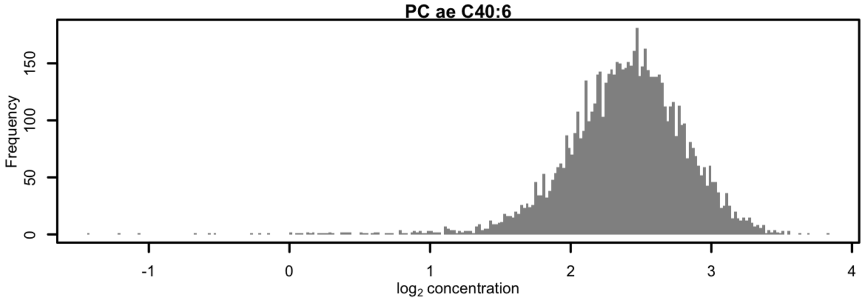

Figure S153: Signal distribution for PC ae C40:6.

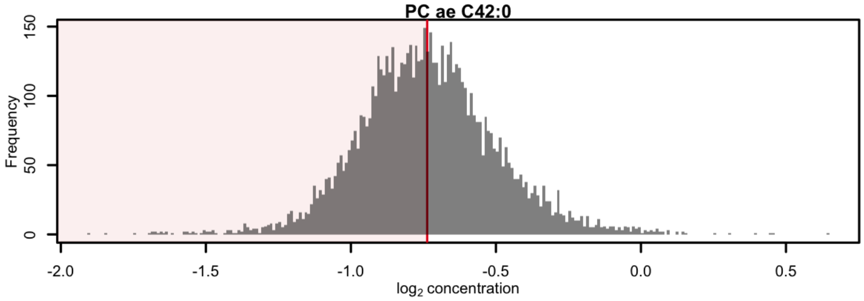

Figure S154: Signal distribution for PC ae C42:0.

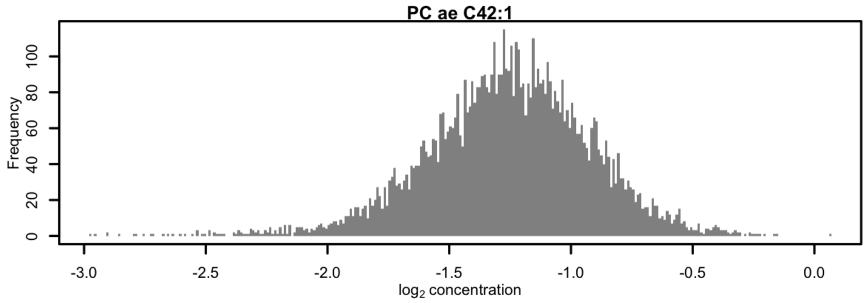

Figure S155: Signal distribution for PC ae C42:1.

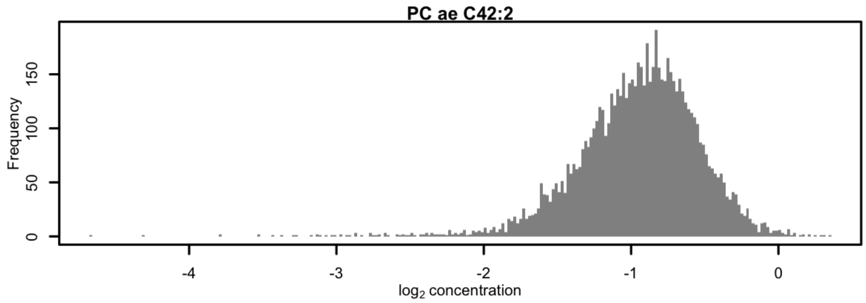

Figure S156: Signal distribution for PC ae C42:2.

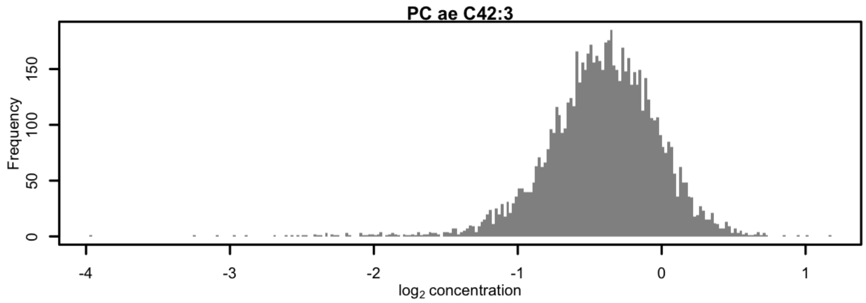

Figure S157: Signal distribution for PC ae C42:3.

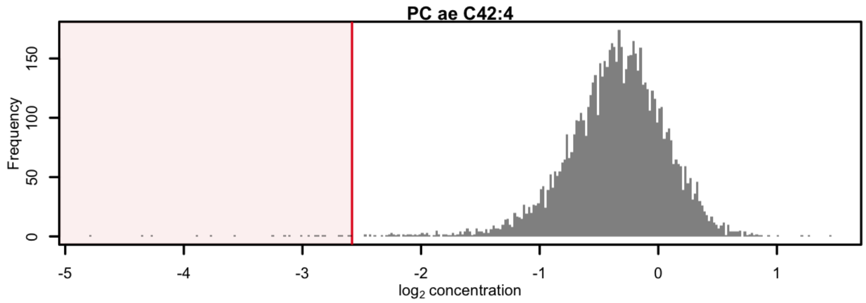

Figure S158: Signal distribution for PC ae C42:4.

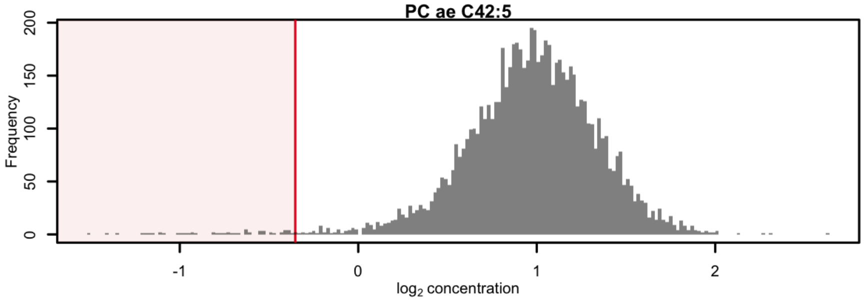

Figure S159: Signal distribution for PC ae C42:5.

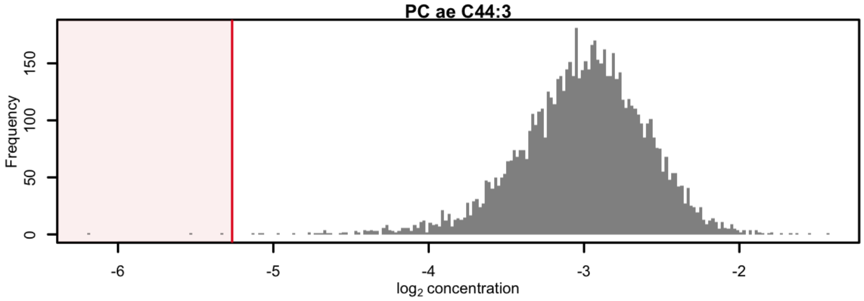

Figure S160: Signal distribution for PC ae C44:3.

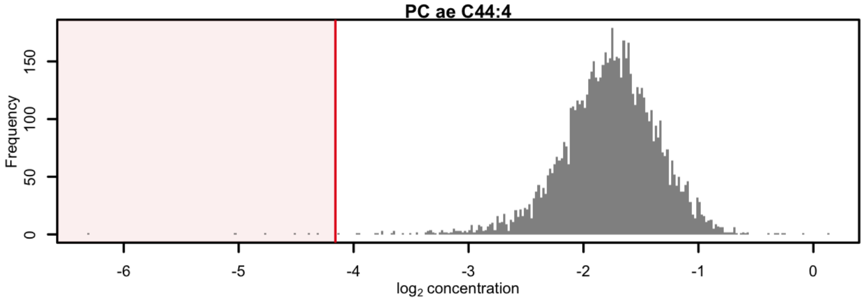

Figure S161: Signal distribution for PC ae C44:4.

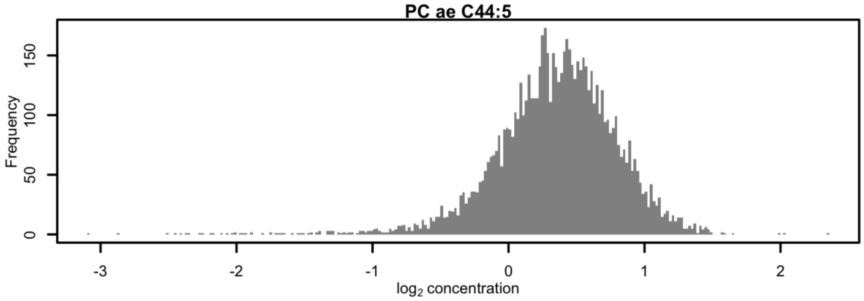

Figure S162: Signal distribution for PC ae C44:5.

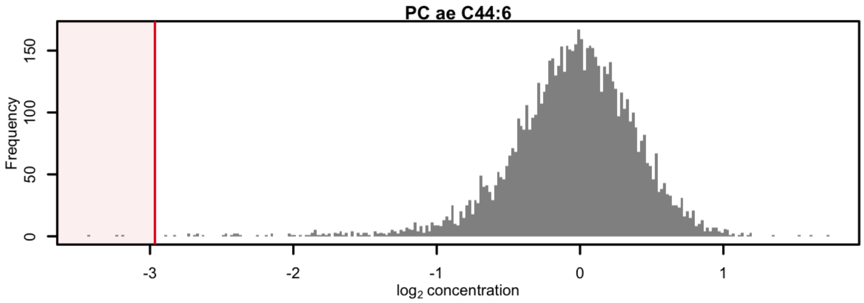

Figure S163: Signal distribution for PC ae C44:6.

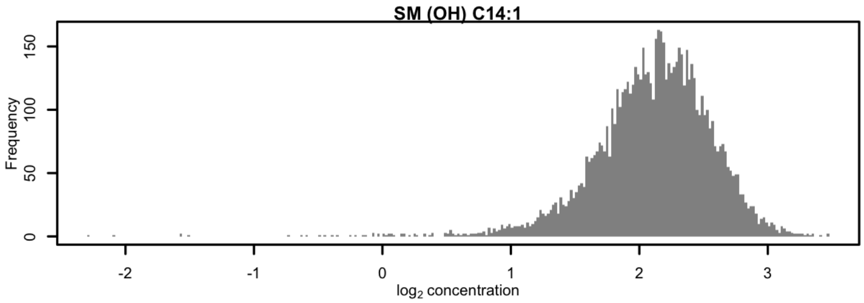

Figure S164: Signal distribution for SM (OH) C14:1.

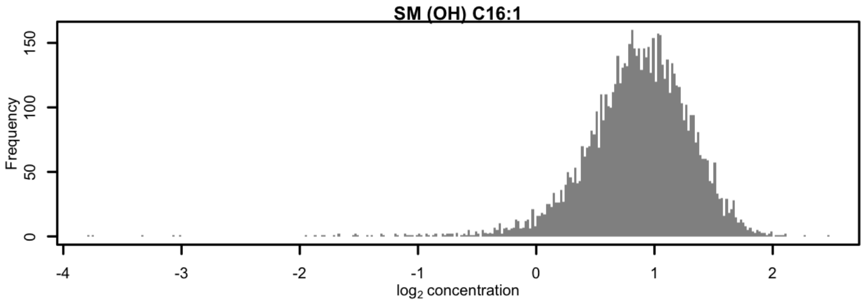

Figure S165: Signal distribution for SM (OH) C16:1.

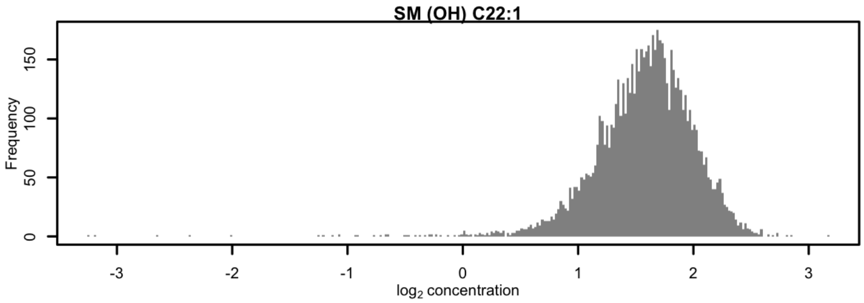

Figure S166: Signal distribution for SM (OH) C22:1.

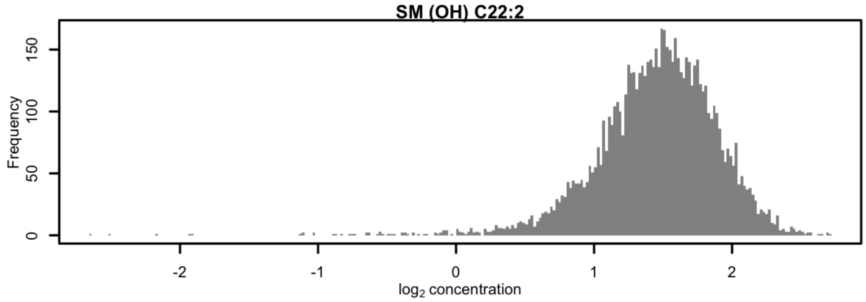

Figure S167: Signal distribution for SM (OH) C22:2.

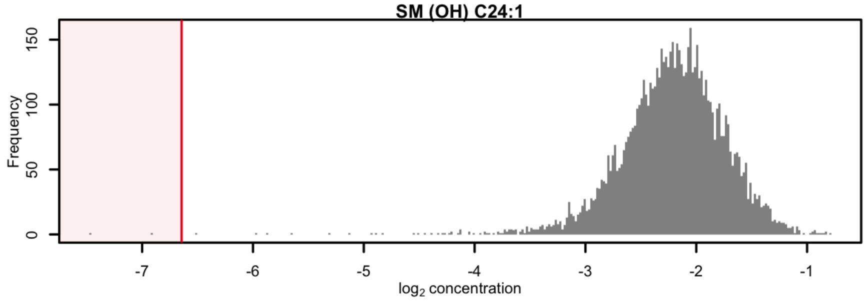

Figure S168: Signal distribution for SM (OH) C24:1.

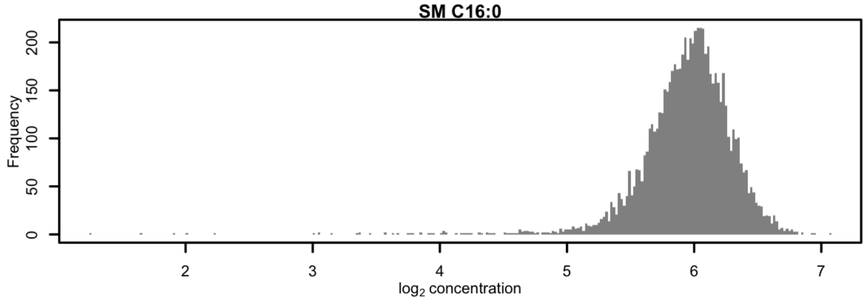

Figure S169: Signal distribution for SM C16:0.

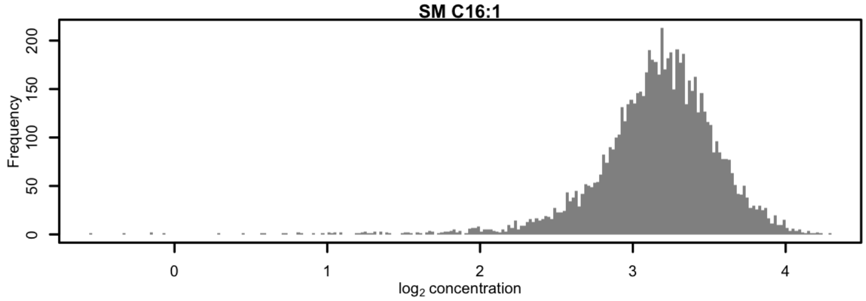

Figure S170: Signal distribution for SM C16:1.

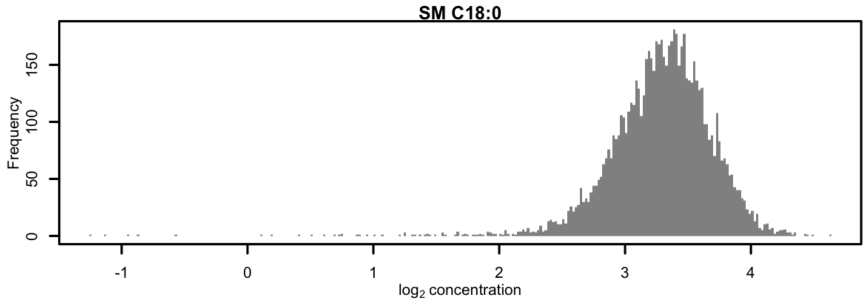

Figure S171: Signal distribution for SM C18:0.

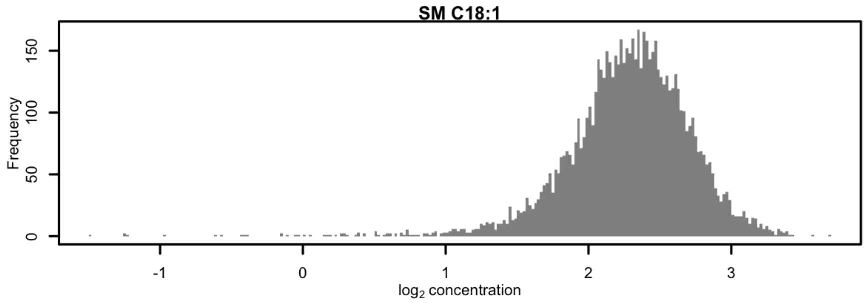

Figure S172: Signal distribution for SM C18:1.

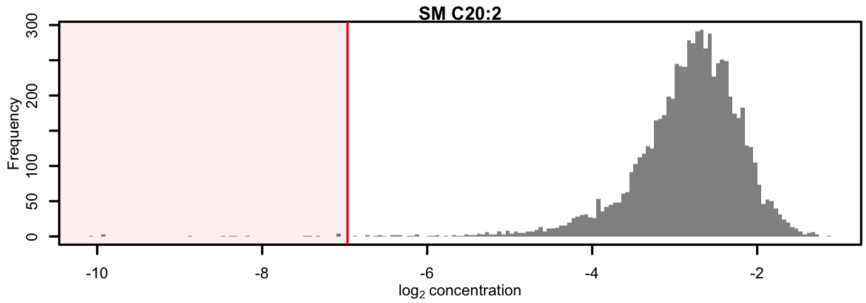

Figure S173: Signal distribution for SM C20:2.

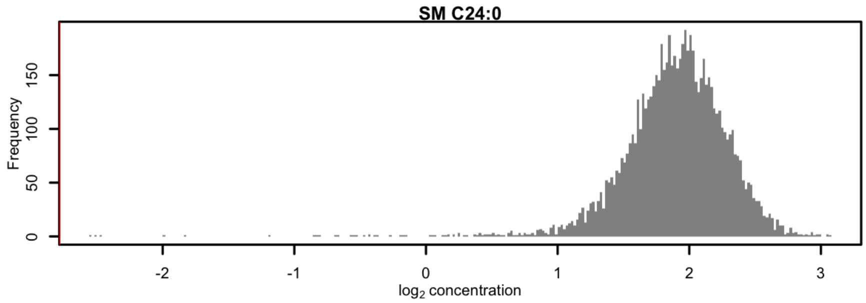

Figure S174: Signal distribution for SM C24:0.

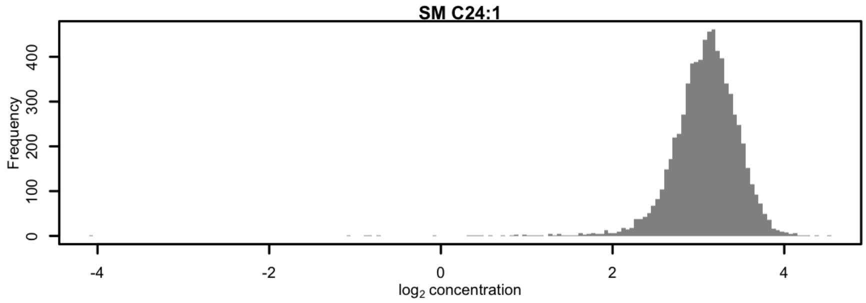

Figure S175: Signal distribution for SM C24:1.

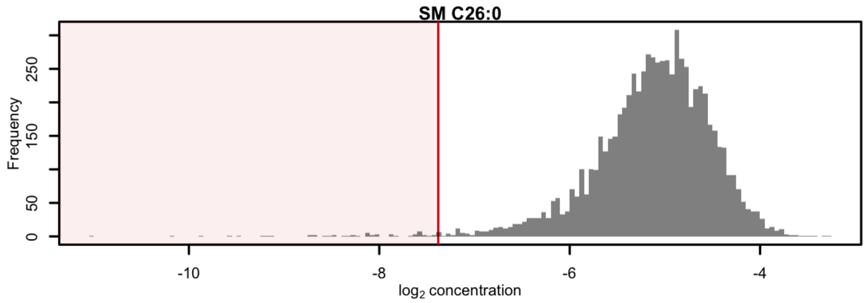

Figure S176: Signal distribution for SM C26:0.

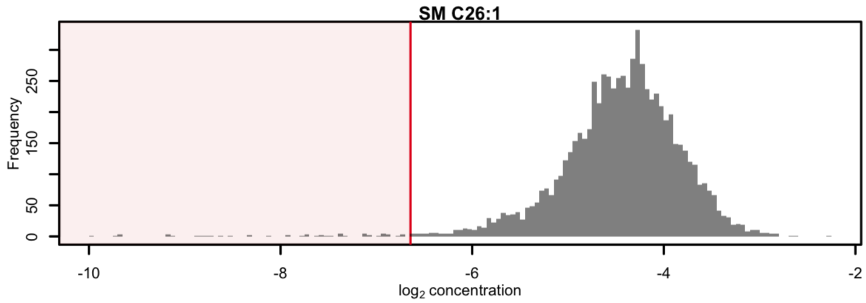

Figure S177: Signal distribution for SM C26:1.

Sex-related metabolites

The metabolites with significant differences in metabolite concentrations between female and male study participants are listed in the table below. All metabolites have an adjusted p-value smaller than 0.05 and a difference in concentrations which is at least twice as large as the coefficient of variation in QC (study pool) samples.

Table S4: Metabolites with significant difference in concentrations between female and male study participants. $coef$ and p_{adj}: coefficient (representing the differential abundance in log2 scale) and p-value adjusted for multiple hypothesis testing. C_{Female} and C_{Male}: average concentration in female and male participants, respectively. Metabolites are ordered by p-value.

$Name$	$Class$	$coef$	p_{adj}	C_{Female}	C_{Male}
Creatinine	biogenic amines	-0.3	0	69	84.9
Ile	aminoacids	-0.318	0	58	73.2
Leu	aminoacids	-0.306	0	118	147
lysoPC a C20:4	GP	-0.456	0	4.43	6.04
C5	acylcarnitines	-0.393	5.99e-311	0.112	0.149
lysoPC a C18:2	GP	-0.443	7.76e-281	18.2	24
Val	aminoacids	-0.223	6.04e-279	201	238
lysoPC a C18:1	GP	-0.347	6.62e-222	12.8	15.8
lysoPC a C20:3	GP	-0.345	1.17e-210	1.78	2.26
C0	acylcarnitines	-0.226	1.13e-185	34.7	41.1
Sarcosine	biogenic amines	-0.393	2.25e-180	1.13	1.48
Pro	aminoacids	-0.296	4.73e-180	151	187
C3	acylcarnitines	-0.292	9.98e-175	0.326	0.408
Met	aminoacids	-0.18	2.83e-172	21.2	24
Trp	aminoacids	-0.159	7.88e-164	58	64.9
SM (OH) C22:2	sphingolipids	0.282	2.05e-157	2.98	2.45
lysoPC a C16:0	GP	-0.204	1.26e-148	53.4	61.2
PC aa C32:3	GP	0.277	9.86e-138	1.49	1.23
t4-OH-Pro	biogenic amines	-0.337	2.04e-132	7.08	8.98
Glu	aminoacids	-0.281	2.92e-123	38.2	48.8
PC ae C32:2	GP	0.233	8.95e-120	2.26	1.92
SM C18:1	sphingolipids	0.242	3.45e-116	5.18	4.45
PC aa C32:2	GP	0.337	1.54e-106	15.3	12.2
Orn	aminoacids	-0.233	5.82e-99	64.2	75.9
PC aa C34:3	GP	0.266	7.78e-98	38.3	31.9
lysoPC a C18:0	GP	-0.217	8.35e-95	16.7	19.3
SM C16:1	sphingolipids	0.202	3.11e-94	9.43	8.26
PC ae C36:2	GP	0.225	1.94e-91	30.2	25.5
SM (OH) C14:1	sphingolipids	0.22	2.48e-88	4.6	3.94
SM (OH) C16:1	sphingolipids	0.214	2.81e-87	1.93	1.67
PC ae C38:3	GP	0.22	3.75e-87	7.07	6.09
PC ae C34:1	GP	0.202	6.58e-82	26.2	22.6
PC aa C36:6	GP	0.272	1.18e-73	1.75	1.46
Tyr	aminoacids	-0.129	9.06e-69	60.3	67
PC aa C30:0	GP	0.236	8.24e-68	3.52	2.98
PC ae C38:0	GP	0.197	3.39e-62	2.95	2.57
Gly	aminoacids	0.134	2.5e-45	251	227
Cit	aminoacids	-0.115	4.61e-43	28.1	30.4
PC aa C34:4	GP	0.205	2.23e-42	5.17	4.58
PC aa C32:1	GP	0.222	1.62e-34	48.1	41.9
alpha-AAA	biogenic amines	-0.517	8.35e-18	0.283	0.422
Serotonin	biogenic amines	0.153	3.17e-11	0.636	0.556

The significant metabolite sums (sum of concentrations for pre-defined metabolite classes) are listed below.

Table S5: Metabolite sums with significant difference in concentrations between female and male study participants. $coef$ and p_{adj}: coefficient (representing the differential abundance in log2 scale) and p-value adjusted for multiple hypothesis testing.Results are ordered by p-value.

Name	*Description*	$coef$	p_{adj}
tBCAA	Sum of branched amino acids	-0.264	0
tEssentAA	Sum of essential amino acids	-0.158	4.54e-248
tAC	Sum of acylcarnitines	-0.189	1.24e-160

Significant metabolite ratios (ratios of concentrations between selected metabolites or metabolite groups) are listed below.

Table S6: Significant metabolite ratios between female and male study participants. $coef$ and p_{adj}: coefficient (representing the difference in ratios in log2 scale) and p-value adjusted for multiple hypothesis testing.Results are ordered by p-value.

Name	*Description*	$coef$	p_{adj}
tLysoPC/tPC	Ratio of lysoglycerophosphocholines to glycerophosphocholines	-0.37	6.34e-294
Fischer_ratio	Fischer ratio	-0.153	1.01e-190
C0/tPC	Ratio of choline and total phosphocholines	-0.33	4.7e-188
Orn/Arg	Ratio of ornithine to arginine	-0.226	2.03e-115
DOPA/Tyr	Ratio of dopamine to tyrosine	0.133	1.38e-68
C2/C0	Ratio of acetylcarnitine to free carnitine	0.196	4.32e-60
tC2C3/C0	Ratio of short chain acetylcanitine to free carnitine	0.182	4.79e-57
Serotonin/Trp	Ratio of serotonin to tryptophan	0.313	3.02e-46

Age-related metabolites

The metabolites which concentrations are significantly related to participant's age are listed below. All metabolites with an adjusted p-value smaller than 0.05 are considered *significant*.

Table S7: Metabolites with significant regression of concentrations on participants' age. $coef$ and p_{adj}: coefficient (representing the difference in abundance for one year difference in age) and p-value adjusted for multiple hypothesis testing. Metabolites are ordered by p-value.

Name	*Class*	$coef$	p_{adj}
Cit	aminoacids	0.0111	0
SDMA	biogenic amines	0.00631	1.05e-176
C18	acylcarnitines	0.00775	1.13e-165
C18:1	acylcarnitines	0.00712	3.12e-153
lysoPC a C17:0	GP	0.00879	2.63e-145
C16:1	acylcarnitines	0.00631	8.19e-142
SM (OH) C16:1	sphingolipids	0.00817	3.29e-131
C6 (C4:1-DC)	acylcarnitines	0.00786	9.18e-128
Orn	aminoacids	0.00815	2.84e-126
SM (OH) C14:1	sphingolipids	0.00805	2.16e-122
C2	acylcarnitines	0.00814	5.38e-108
C16	acylcarnitines	0.00607	1.36e-105
PC ae C30:2	GP	0.00717	5.36e-104
H1	sugars	0.00316	4.84e-102

$Name$	$Class$	$coef$	p_{adj}
PC aa C36:5	GP	0.0106	7.85e-102
lysoPC a C18:0	GP	0.00693	2.07e-101
ADMA	biogenic amines	0.0051	1.16e-93
PC aa C28:1	GP	0.00721	3.94e-93
PC ae C34:0	GP	0.00742	2.66e-92
PC ae C36:1	GP	0.00649	4.8e-85
SM C16:1	sphingolipids	0.00585	2.14e-83
Serotonin	biogenic amines	-0.0126	1.38e-80
Trp	aminoacids	-0.00337	6.15e-79
C3	acylcarnitines	0.00594	4.63e-78
lysoPC a C28:1	GP	0.00624	2.12e-77
SM C16:0	sphingolipids	0.00519	1.61e-76
C16:1-OH	acylcarnitines	0.00448	7.47e-75
PC aa C40:5	GP	0.00676	1.9e-74
C8	acylcarnitines	0.00661	2.03e-73
C0	acylcarnitines	0.00429	6.5e-73
C9	acylcarnitines	0.00479	3.87e-70
Gln	aminoacids	0.00302	1.01e-69
C10	acylcarnitines	0.00727	1.88e-68
C12:1	acylcarnitines	0.00528	2.34e-68
PC ae C40:2	GP	0.00558	3.54e-68
C14:1-OH	acylcarnitines	0.00395	1.54e-64
PC aa C38:5	GP	0.0057	1.24e-63
C14:1	acylcarnitines	0.00738	1.04e-61
SM (OH) C24:1	sphingolipids	0.00605	1.13e-61
Kynurenine	biogenic amines	0.00445	2.12e-61
Thr	aminoacids	-0.004	1.7e-60
SM (OH) C22:2	sphingolipids	0.00528	8.19e-60
C12	acylcarnitines	0.00518	1.17e-59
C14	acylcarnitines	0.00512	1.51e-59
His	aminoacids	-0.00242	2.88e-59
C10:1	acylcarnitines	0.00515	6.26e-57
PC ae C36:2	GP	0.00516	3.31e-51
SM (OH) C22:1	sphingolipids	0.00487	9.88e-50
lysoPC a C24:0	GP	0.00373	8.2e-48
C10:2	acylcarnitines	0.00235	1.08e-47
lysoPC a C16:0	GP	0.00348	9.85e-47
PC aa C40:6	GP	0.00567	2.42e-44
PC aa C32:0	GP	0.00418	1.22e-42
C4	acylcarnitines	0.00574	5.02e-42
lysoPC a C18:1	GP	0.00451	1.6e-41
SM C18:1	sphingolipids	0.00441	2.4e-41
Asn	aminoacids	-0.00232	7.2e-41
Gly	aminoacids	0.00393	9.9e-41
Tyr	aminoacids	0.00303	2.81e-40
PC ae C40:6	GP	0.00445	5.65e-40
SM C18:0	sphingolipids	0.00419	6.72e-40
C14:2	acylcarnitines	0.00529	8.69e-40
C5:1-DC	acylcarnitines	0.00278	8.61e-37
PC ae C42:2	GP	0.00411	2.83e-36
PC aa C32:3	GP	0.00431	6.11e-36
Arg	aminoacids	0.00261	1.99e-35

Name	*Class*	*coef*	p_{adj}
PC ae C38:3	GP	0.00428	8.56e-35
PC aa C36:1	GP	0.0045	2.41e-34
PC ae C38:0	GP	0.00439	1.46e-32
PC aa C30:0	GP	0.00488	1.42e-30
Glu	aminoacids	0.00428	1.49e-30
PC ae C34:1	GP	0.00378	1.94e-30
PC aa C40:3	GP	0.00341	6.72e-30
SM C26:0	sphingolipids	0.00615	8.37e-29
PC aa C36:6	GP	0.00517	4.58e-28
PC ae C32:2	GP	0.00342	1.27e-27
PC ae C38:2	GP	0.0039	3.58e-24
C4:1	acylcarnitines	0.00221	4.61e-24
PC aa C36:2	GP	0.00327	4.47e-23
lysoPC a C26:1	GP	0.00281	4.6e-22
lysoPC a C16:1	GP	0.00327	9.3e-22
PC aa C42:6	GP	0.00317	9.92e-22
C5-DC (C6-OH)	acylcarnitines	0.00262	4.05e-21
PC ae C40:1	GP	0.00313	3.06e-20
PC ae C36:0	GP	0.00316	3.46e-20
Asp	aminoacids	-0.00279	1.33e-19
PC aa C38:3	GP	0.00342	2.6e-19
SM C26:1	sphingolipids	0.00537	4.31e-19
SM C24:0	sphingolipids	0.00288	4.69e-19
PC ae C40:5	GP	0.00283	7.63e-19
Ile	aminoacids	-0.00189	1.15e-18
PC aa C42:2	GP	0.00273	1.58e-18
PC aa C38:4	GP	0.0032	3.87e-18
PC aa C34:3	GP	0.00352	4.56e-18
C5-OH (C3-DC-M)	acylcarnitines	0.00173	5.25e-18
PC aa C42:5	GP	0.00299	8.53e-17
Sarcosine	biogenic amines	0.0036	1.43e-16
PC aa C36:0	GP	0.00334	1.82e-16
C5-M-DC	acylcarnitines	0.00187	2.63e-15
PC ae C40:3	GP	0.00238	5.25e-15
Met	aminoacids	-0.00161	1.19e-14
Spermidine	biogenic amines	-0.00166	3.03e-14
Lys	aminoacids	0.00165	5.07e-14
t4-OH-Pro	biogenic amines	-0.00335	9.88e-14
PC aa C34:1	GP	0.00291	1.06e-13
PC aa C40:2	GP	0.00269	1.24e-13
PC aa C40:4	GP	0.00284	2.88e-13
C16-OH	acylcarnitines	0.00128	1.5e-12
Leu	aminoacids	-0.00136	1.7e-11
Ser	aminoacids	-0.00138	4.45e-11
C5	acylcarnitines	0.00221	7.04e-11
PC ae C38:4	GP	0.00227	8.89e-11
PC aa C38:0	GP	0.00252	1.19e-10
PC aa C40:1	GP	0.00162	6.36e-10
SM C24:1	sphingolipids	0.00214	6.46e-10
Val	aminoacids	-0.00125	1.5e-09
PC ae C38:6	GP	0.00228	4.16e-09
PC ae C42:1	GP	0.00171	7e-09

Name	*Class*	*coef*	p_{adj}
PC aa C32:1	GP	0.00354	1e-08
lysoPC a C20:3	GP	0.00215	1.4e-08
lysoPC a C14:0	GP	0.00091	5.16e-08
PC ae C44:5	GP	-0.00212	7.43e-08
C3-DC (C4-OH)	acylcarnitines	0.00228	7.53e-08
PC aa C42:4	GP	0.00191	1.38e-07
PC ae C36:5	GP	0.00217	3.74e-07
PC aa C38:6	GP	0.00231	3.86e-07
Taurine	biogenic amines	-0.00131	4.22e-07
PC ae C30:0	GP	0.00214	6.24e-07
PC aa C24:0	GP	0.0026	2.05e-06
lysoPC a C20:4	GP	0.00182	2.9e-06
PC ae C40:4	GP	0.00165	4.21e-06
PC aa C42:1	GP	0.00172	4.76e-06
C5:1	acylcarnitines	0.00125	6.25e-06
C6:1	acylcarnitines	0.00115	7.23e-06
C14:2-OH	acylcarnitines	0.00143	8.23e-06
PC ae C42:3	GP	0.00146	0.000107
C12-DC	acylcarnitines	-0.000528	0.000118
PC ae C44:3	GP	0.00154	0.000146
PC ae C38:5	GP	0.00153	0.000165
PC aa C34:4	GP	0.00206	0.000712
SM C20:2	sphingolipids	0.00285	0.000915
PC ae C32:1	GP	0.00141	0.000954
PC aa C26:0	GP	0.000645	0.0026
PC aa C42:0	GP	0.00128	0.00585
PC ae C42:0	GP	0.000727	0.00986
PC aa C36:3	GP	0.00132	0.0116
PC aa C32:2	GP	0.0017	0.041
Creatinine	biogenic amines	0.000678	0.0432

The table below lists the significant sums of concentrations for metabolite groups.

Table S8: Metabolite sums with significant regression of concentrations on participants' age. *coef* and p_{adj}: coefficient (representing the difference in abundance for one year difference in age) and p-value adjusted for multiple hypothesis testing. Results are ordered by p-value.

Name	*Description*	*coef*	p_{adj}
tAC	Sum of acylcarnitines	0.00487	7.73e-114
tSM	Sum all sphingomyelins	0.00496	3.04e-72
tSFA_PC	Sum of saturated glycerophosphocholine without Lyso	0.00414	3.51e-47
tNonEssentAA	Sum of non-essentila amino acids (Biocrates; without Cys)	0.00196	4.28e-42
tACDC	Sum of dicarboxy-acylcarnitines	0.00151	3.87e-29
tEssentAA	Sum of essential amino acids	-0.00124	5.18e-18
tSelectedGlucoAA	Sum of selected glucogenic amino acids (alanine, glycine, serine)	0.00144	1.21e-14
tBCAA	Sum of branched amino acids	-0.00139	3.72e-14
tGP	Sum of all LysoPC and PC	0.00223	5.09e-14
tPUFA_PC	Sum of polyunsaturated glycerophosphocholine without Lyso	0.00198	1.11e-10

The table below lists the significant ratios of concentrations between metabolites and metabolite groups.

Table S9: Metabolite ratios with significant regression on participants' age. *coef* and p_{adj}: coefficient (representing the difference in ratio for one year difference in age) and p-value adjusted for multiple hypothesis testing. Results are ordered by p-value.

Name	*Description*	*coef*	p_{adj}
Cit/Arg	Ratio of citrulline to arginine	0.00852	2.86e-214
Kynurenine/Trp	Ratio of kyneurine to tryptophan	0.00782	3.76e-199
Orn/Arg	Ratio of ornithine to arginine	0.00577	2.43e-80
Fischer_ratio	Fischer ratio	-0.00293	1.21e-76
Putrescine/Orn	Ratio of putrescine to ornithine	-0.00754	3.75e-73
tACDC/tAC	Ratio of dicarboxy-acylcarnitines to total acylcarnitine	-0.00336	2.32e-54
tPUFA_PC/tSFA_PC	Ratio of polyunsaturated to saturated glycerophosphocholine	-0.00216	4.16e-53
Tyr/Phe	Ratio of thyrosine to phenylalanine	0.00296	6.73e-45
Serotonin/Trp	Ratio of serotonin to tryptophan	-0.00918	6.91e-42
SDMA/Arg	Ratio of symmetrically dimethylated arginine to total unmodified arginine	0.00366	6.5e-37
tADMASDMA/Arg	Ratio of total dymethylated arginine to total unmodified arginine	0.00312	1.12e-31
DOPA/Tyr	Ratio of dopamine to tyrosine	-0.00274	6.46e-31
tC2C3/C0	Ratio of short chain acetylcanitine to free carnitine	0.00373	1.53e-25
C2/C0	Ratio of acetylcarnitine to free carnitine	0.00385	7.88e-25
Cit/Orn	Ratio of citrulline to ornithine	0.00274	8.4e-23
ADMA/Arg	Ratio of asymmetrically dimethylated arginine to total unmodified arginine	0.00239	2.02e-17
tC16C18/C0	Ratio of C16 C18 to C0	0.00234	6.58e-15
Spermidine/Putrescine	Ratio of spermidine to putrescine	-0.00249	3.39e-14
Met-SO/Met	Ratio of sulfoxide methionine to total unmodified methionine	0.00816	7.45e-13
C0/tPC	Ratio of choline and total phosphocholines	0.00204	2.82e-08
tMUFA_PC/tSFA_PC	Ratio of monounsaturated to saturated glycerophosphocholines	-0.000931	5.37e-05
Spermine/Spermidine	Ratio of spermine to spermidine	0.00119	0.000369
tLysoPC/tPC	Ratio of lysoglycerophosphocholines to glycerophosphocholines	0.00121	0.000927

Metabolites related to body mass index

Metabolites with significant differences in concentrations between body mass index (BMI) categories 1 (BMI < 18.5), 3 (25 <= BMI < 30) and 4 (BMI > 30) to the *normal* category 2 (18.5 <= BMI < 25) are listed in the tables below. Metabolites with an adjusted p-value smaller than 0.05 and a difference in concentrations larger than 2 times the coefficient of variation in QC (study pool) samples are considered significant.

Table S10: Metabolites with significant differences in concentrations between study participants with a BMI < 18.5 (BMI1) and participants with BMI between 18.5 and 25 (BMI2). *coef* and p_{adj}: coefficient (representing the difference in abundance in log2 scale) and p-value adjusted for multiple hypothesis testing. C_{BMI1} and C_{BMI2}: average concentration for the compared categories. Metabolites are ordered by p-value.

Name	*Class*	*coef*	p_{adj}	C_{BMI1}	C_{BMI2}
Tyr	aminoacids	-0.129	0.00182	52.3	59.3

Table S11: Metabolites with significant differences in concentrations between study participants with a BMI between 25 and 30 (BMI3) and participants with a BMI between 18.5 and 25 (BMI2). $coef$ and p_{adj}: coefficient (representing the difference in abundance in log2 scale) and p-value adjusted for multiple hypothesis testing. C_{BMI3} and C_{BMI2}: average concentration for the compared categories. Metabolites are ordered by p-value.

$Name$	$Class$	$coef$	p_{adj}	C_{BMI3}	C_{BMI2}
Glu	aminoacids	0.332	4.66e-133	47.8	35.6
lysoPC a C18:2	GP	-0.231	6.41e-64	20.4	22.3
lysoPC a C18:1	GP	-0.194	6.43e-57	14	14.9
PC aa C38:3	GP	0.164	3.66e-33	64.2	57.3
lysoPC a C17:0	GP	-0.148	2.55e-31	1.22	1.27
C3	acylcarnitines	0.122	2.25e-24	0.387	0.33
Serotonin	biogenic amines	-0.167	3.1e-10	0.55	0.677

Table S12: Metabolites with significant differences in abundances between study participants with a BMI > 30 (BMI4) and participants with BMI between 18.5 and 25 (BMI2). $coef$ and p_{adj}: coefficient (representing the difference in abundance in log2 scale) and p-value adjusted for multiple hypothesis testing. C_{BMI4} and C_{BMI2}: average concentration for the compared categories. Metabolites are ordered by p-value.

$Name$	$Class$	$coef$	p_{adj}	C_{BMI4}	C_{BMI2}
Glu	aminoacids	0.667	2.53e-310	59.4	35.6
lysoPC a C18:2	GP	-0.452	3.52e-146	16.9	22.3
Val	aminoacids	0.223	1.48e-139	240	205
lysoPC a C18:1	GP	-0.376	2.42e-128	12.1	14.9
Ile	aminoacids	0.229	3.98e-123	71.6	60.6
lysoPC a C17:0	GP	-0.354	1.68e-108	1.07	1.27
Leu	aminoacids	0.189	2.11e-102	142	123
Tyr	aminoacids	0.228	2.53e-102	71.7	59.3
H1	sugars	0.124	1.45e-72	4796	4274
PC aa C38:3	GP	0.301	3.6e-68	71.8	57.3
C3	acylcarnitines	0.246	7.22e-61	0.417	0.33
C5	acylcarnitines	0.233	9.65e-57	0.145	0.118
PC ae C34:3	GP	-0.283	1.42e-56	18.9	23.2
Phe	aminoacids	0.137	4.21e-56	67.3	60.8
Gly	aminoacids	-0.214	1.69e-54	220	250
Kynurenine	biogenic amines	0.192	7.63e-52	2.94	2.46
Ala	aminoacids	0.166	8.37e-52	366	324
Asp	aminoacids	0.206	4.39e-49	15.3	13.7
PC ae C36:2	GP	-0.217	4.28e-41	25.8	29.3
lysoPC a C16:0	GP	-0.142	3.12e-35	53.9	57.2
PC aa C38:4	GP	0.208	3.36e-35	163	138
lysoPC a C18:0	GP	-0.186	1.52e-33	16.9	17.9
Asn	aminoacids	-0.093	8.82e-30	42.8	46.3
Cit	aminoacids	-0.132	5.06e-27	28.8	28.8
Serotonin	biogenic amines	-0.323	2.03e-24	0.488	0.677
PC aa C34:4	GP	0.198	9.89e-19	5.36	4.66
PC aa C32:1	GP	0.235	3.55e-18	51.2	43
lysoPC a C20:4	GP	-0.139	9.45e-18	4.82	5.08
alpha-AAA	biogenic amines	0.695	3e-15	0.48	0.28

The table below lists the significant sums of concentrations for metabolite groups (no significant sums were

found for the comparison of BMI categories 1 or 3 against 2).

Table S13: Metabolite sums with significant differences in abundances between study participants with a BMI > 30 (BMI4) and participants with BMI between 18.5 and 25 (BMI2). $coef$ and p_{adj}: coefficient (representing the difference in abundance in log2 scale) and p-value adjusted for multiple hypothesis testing. Results are ordered by p-value.

Name	*Description*	*coef*	p_{adj}
tBCAA	Sum of branched amino acids	0.213	1.31e-137
tEssentAA	Sum of essential amino acids	0.126	1.16e-80

The table below lists the significant ratios between metabolite (or metabolite group) concentrations between BMI categories (1, 3 and 4 against 2).

Table S14: Significant metabolite ratios between study participants with a BMI < 18.5 (BMI1) and participants with BMI between 18.5 and 25 (BMI2). $coef$ and p_{adj}: coefficient (representing the difference in ratios in log2 scale) and p-value adjusted for multiple hypothesis testing. Results are ordered by p-value.

Name	*Description*	*coef*	p_{adj}
DOPA/Tyr	Ratio of dopamine to tyrosine	0.129	0.000602
Tyr/Phe	Ratio of thyrosine to phenylalanine	-0.109	0.00162

Table S15: Significant metabolite ratios between study participants with a BMI between 25 and 30 (BMI3) and participants with BMI between 18.5 and 25 (BMI2). $coef$ and p_{adj}: coefficient (representing the difference in ratios in log2 scale) and p-value adjusted for multiple hypothesis testing. Results are ordered by p-value.

Name	*Description*	*coef*	p_{adj}
Serotonin/Trp	Ratio of serotonin to tryptophan	-0.186	5.84e-13

Table S16: Significant metabolite ratios between study participants with a BMI > 30 (BMI4) and participants with BMI between 18.5 and 25 (BMI2). $coef$ and p_{adj}: coefficient (representing the difference of ratios in log2 scale) and p-value adjusted for multiple hypothesis testing. Results are ordered by p-value.

Name	*Description*	*coef*	p_{adj}
DOPA/Tyr	Ratio of dopamine to tyrosine	-0.226	4.29e-93
tLysoPC/tPC	Ratio of lysoglycerophosphocholines to glycerophosphocholines	-0.234	3.02e-61
Cit/Arg	Ratio of citrulline to arginine	-0.16	6.72e-37
Serotonin/Trp	Ratio of serotonin to tryptophan	-0.378	1.65e-32

Menopause associated metabolites

Metabolites associated with menopause identified on the full data set are shown below.

Table S17: Metabolites with significant difference in concentrations between post- and premenopausal women. $coef$ and p_{adj}: coefficient (representing the differential abundance in log2 scale) and p-value adjusted for multiple hypothesis testing. ES: effect size. CV_{QC}: coefficient of variation (in %) calculated across quality control samples. Metabolites are ordered by p-value.

$Name$	$Class$	$coef$	p_{adj}	ES	CV_{QC}
Glu	aminoacids	0.231	5.01e-17	0.429	3.76
Asp	aminoacids	0.176	6.22e-14	0.436	5.46
t4-OH-Pro	biogenic amines	0.21	5.9e-11	0.398	7.07
C3	acylcarnitines	0.146	3.72e-09	0.33	4.27
C2	acylcarnitines	0.14	5e-06	0.289	2.33
Sarcosine	biogenic amines	0.148	0.000157	0.278	4.85

Metabolites identified by the age-restricted analysis are shown below.

Table S18: Metabolites with significant difference in concentrations between post- and premenopausal women identified for women aged 49-57. $coef$ and p_{adj}: coefficient (representing the differential abundance in log2 scale) and p-value adjusted for multiple hypothesis testing. ES: effect size. CV_{QC}: coefficient of variation (in %) calculated across quality control samples. Metabolites are ordered by p-value.

$Name$	$Class$	$coef$	p_{adj}	ES	CV_{QC}
C2	acylcarnitines	0.161	0.000927	0.336	2.33
Glu	aminoacids	0.111	0.0156	0.222	3.76
Sarcosine	biogenic amines	0.164	0.0156	0.264	4.85

Significant sums of concentrations for groups of metabolites from the age-restricted analysis are shown below.

Table S19: Metabolite sums with significant difference in concentrations between pre- and postmenopausal women. $coef$ and p_{adj}: coefficient (representing the differential abundance in log2 scale) and p-value adjusted for multiple hypothesis testing.Results are ordered by p-value. Analysis performed on women aged 49 - 57.

$Name$	$Description$	$coef$	p_{adj}
tAC	Sum of acylcarnitines	0.145	1.75e-11

Significant ratios of concentrations between metabolites or groups of metabolites from the age-restricted analysis are shown below.

Table S20: Significant metabolite ratios between pre- and postmenopausal women. $coef$ and p_{adj}: coefficient (representing the difference in ratios in log2 scale) and p-value adjusted for multiple hypothesis testing.Results are ordered by p-value. Analysis performed on women aged 49 - 57.

$Name$	$Description$	$coef$	p_{adj}
tACDC/tAC	NA	-0.123	1.14e-07
tC16C18/C0	NA	-0.132	9.22e-05
C0/tPC	NA	0.145	0.000164
Orn/Arg	NA	0.0975	0.0172

Metabolites related to food items

Supplementary Table S21 (file *Table_S21-food-item-average-servings.xlsx*: Average servings per week of food items for bottom and top 20% of individuals with lowest and highest consumption. Columns $meanQ1$, $sdQ1$, $meanQ5$ and $sdQ5$: average and standard deviation for (energy intake-adjusted) servings per week for bottom and top 20% of individuals with lowest and highest consumption of the respective food item.

Supplementary Table S22 (file *Table_S22-food-item-results.xlsx*): Pairwise relationships between all metabolites and food items. Column $coefQ5vsQ1$: log2 difference in metabolite concentration between top and bottom 20% of individuals with the highest and lowest consumption of the respective food item. Significance for this relationship is provided in columns $p-valueQ5vsQ1$ and $p-adjQ5vsQ1$ (Bonferroni adjusted p-values). $significantQ5vsQ1$: whether the relationship was considered significant. $meanQ1$, $sdQ1$, $meanQ5$ and $sdQ5$ average and standard deviation for (energy intake-adjusted) servings per week for bottom and top 20% of individuals with lowest and highest consumption of the respective food item.

Table S23: Significant relationships between metabolites and food items. Column $coef$: (log2) difference in metabolite concentration between the top and bottom 20% of individuals with the highest and lowest consumption of the food item. Column p_{adj} Bonferroni adjusted p-value. Columns $Q5$ and $Q1$: average (total energy intake adjusted) servings per week for top and bottom 20% of individuals with highest respectively lowest consumption of the food item.

$Metabolite$	$Fooditem$	$coef$	p_{adj}	$Q5$	$Q1$
PC ae C36:4	processed_meat_sw	0.293	1.12e-21	9.83	0.839
PC ae C36:4	pork_sw	0.28	8.79e-20	2.5	0.00155
PC ae C30:0	butter_sw	0.277	1.56e-17	11.7	0.102
PC ae C34:0	butter_sw	0.274	2.5e-17	11.7	0.102
PC aa C38:6	fish_sw	0.281	8.2e-17	4.42	0.325
SM (OH) C16:1	butter_sw	0.232	2.26e-16	11.7	0.102
SM (OH) C14:1	butter_sw	0.234	2.5e-15	11.7	0.102
lysoPC a C17:0	butter_sw	0.264	4.81e-15	11.7	0.102
PC ae C36:2	poultry_sw	-0.234	7.63e-14	2.86	0.0619
PC ae C36:1	poultry_sw	-0.223	2.36e-13	2.86	0.0619
lysoPC a C17:0	poultry_sw	-0.25	8.46e-13	2.86	0.0619
PC aa C38:0	fish_sw	0.226	1.14e-12	4.42	0.325
PC aa C40:6	fish_sw	0.25	6.33e-12	4.42	0.325
PC ae C38:5	pork_sw	0.186	1.76e-10	2.5	0.00155
PC ae C36:2	butter_sw	0.206	2.02e-10	11.7	0.102
t4-OH-Pro	processed_meat_sw	0.259	1.19e-09	9.83	0.839
PC ae C38:5	processed_meat_sw	0.178	3.79e-09	9.83	0.839
PC ae C36:4	beef_sw	0.203	4.12e-09	5.82	0.881
PC ae C36:5	pork_sw	0.213	6.01e-09	2.5	0.00155
PC ae C36:5	beef_sw	0.212	7.71e-09	5.82	0.881
PC aa C38:4	processed_meat_sw	0.197	1.97e-08	9.83	0.839
PC ae C36:5	processed_meat_sw	0.201	1.26e-07	9.83	0.839
t4-OH-Pro	beef_sw	0.234	1.38e-07	5.82	0.881
PC aa C38:4	pork_sw	0.185	3.28e-07	2.5	0.00155
PC aa C34:1	wine_sw	0.2	4.52e-07	7.28	0.000841
PC aa C28:1	butter_sw	0.17	9.96e-07	11.7	0.102
PC aa C40:5	fish_sw	-0.181	1.51e-06	4.42	0.325
PC aa C34:1	beer_sw	0.205	2.29e-06	4.45	0.000734
lysoPC a C17:0	sugar_sw	0.187	8.58e-06	20.3	0.846
PC ae C38:6	pork_sw	0.161	2.15e-05	2.5	0.00155
Gly	pork_sw	-0.14	9.52e-05	2.5	0.00155
t4-OH-Pro	pork_sw	0.198	1e-04	2.5	0.00155

$Metabolite$	$Fooditem$	$coef$	p_{adj}	$Q5$	$Q1$
PC aa C32:1	beer_sw	0.29	0.000114	4.45	0.000734
PC aa C36:4	pork_sw	0.156	0.000238	2.5	0.00155
PC aa C34:3	fish_sw	-0.172	0.000334	4.42	0.325
Gly	processed_meat_sw	-0.135	0.000425	9.83	0.839
PC aa C36:5	wine_sw	0.222	0.000473	7.28	0.000841
PC aa C38:6	bread_sw	-0.169	0.00073	18.6	3.57
PC aa C38:6	wine_sw	0.167	0.000994	7.28	0.000841
lysoPC a C20:3	fish_sw	-0.148	0.00117	4.42	0.325
lysoPC a C17:0	softdrinks_sw	-0.163	0.00184	9.16	0.000713
PC aa C36:4	beer_sw	0.159	0.00202	4.45	0.000734
lysoPC a C17:0	margarines_sw	-0.153	0.00282	2.77	0.000686
PC aa C38:4	pome_fruit_sw	-0.142	0.00621	10.5	0.315
Gly	wine_sw	-0.124	0.00752	7.28	0.000841
PC aa C36:5	potato_sw	-0.194	0.00761	5.57	1.35
lysoPC a C17:0	beer_sw	-0.16	0.0108	4.45	0.000734
PC aa C36:5	poultry_sw	-0.191	0.0131	2.86	0.0619
lysoPC a C18:1	processed_meat_sw	-0.137	0.0213	9.83	0.839
lysoPC a C18:1	poultry_sw	-0.136	0.0253	2.86	0.0619
Serotonin	tea_herbal_sw	0.258	0.0254	9.71	0.000889
PC aa C32:1	wine_sw	0.221	0.0342	7.28	0.000841

Seasonality of food items

To investigate a potential seasonality in the participants' reporting of food item consumption, we performed a logistic regression analysis regressing (binarized) food item frequencies (servings per week) on age, sex, fasting status, BMI and season of participation. Food items were binarized by the median (0 and 1 for values below or above the median serving per week of a food item). Resulting p-values were adjusted for multiple hypothesis testing using Bonferroni's method.

Table S24: Seasonality of food items. Shown are the top 10 food items with the largest differences in reported frequencies for participants being sampled in Winter compare to those participating in Summer. Columns $coef$ and p_{adj} contain the coefficient and (Bonferroni adjusted) p-value from the logistic regression analysis for the difference between Winter and Summer. The remaining columns contain the median servings per week for each food item reported by participants in the respective season.

$Fooditem$	$coef$	p_{adj}	$Winter$	$Fall$	$Spring$	$Summer$
lard	0.542	0.000584	0.00101	0.00105	0.00109	0.00111
othermilk	0.501	0.00273	0.876	0.88	1.18	1.29
nightshade_veg	0.464	0.0101	3.23	3.19	3.43	4.1
tea_herbal	-0.406	0.0677	0.776	0.676	0.622	0.571
citrus_fruit	-0.398	0.0814	4.06	3.02	3.58	3.24
pork	0.35	0.253	0.589	0.594	0.656	0.661
veg_tot	0.347	0.318	30.3	30.7	32.5	35
offal	0.323	0.428	0.00104	0.00109	0.00113	0.00113
stone_fruit	0.332	0.431	2.97	2.59	3.66	3.92
other_vet	0.316	0.608	6.09	6.07	7.25	7.47

Medication overview

Table S25: ATC level 2 medications with a frequency larger than 50. Columns *count*, *female* and *male* list the total number, number of female or male study paricipants with a prescription for the indicated medication. Columns *age* and *BMI* contain the average age and BMI.

ATC	Category	count	female	male	age	BMI
C09	AGENTS ACTING ON THE RENIN-ANGIOTENSIN SYSTEM	745	369	376	65	30
G03	SEX HORMONES AND MODULATORS OF THE GENITAL SYSTEM	642	642	0	31	24
B01	ANTITHROMBOTIC AGENTS	485	197	288	69	28
H03	THYROID THERAPY	483	411	72	56	27
C10	LIPID MODIFYING AGENTS	395	182	213	67	28
C07	BETA BLOCKING AGENTS	315	157	158	66	29
N06	PSYCHOANALEPTICS	298	227	71	57	27
A02	DRUGS FOR ACID RELATED DISORDERS	201	110	91	61	27
C08	CALCIUM CHANNEL BLOCKERS	177	92	85	68	29
N05	PSYCHOLEPTICS	140	100	40	64	28
A12	MINERAL SUPPLEMENTS	138	131	7	64	25
A10	DRUGS USED IN DIABETES	121	57	64	65	30
G02	OTHER GYNECOLOGICALS	104	104	0	35	24
G04	UROLOGICALS	100	4	96	72	28
M01	ANTIINFLAMMATORY AND ANTIRHEUMATIC PRODUCTS	93	54	39	56	27
J01	ANTIBACTERIALS FOR SYSTEMIC USE	76	47	29	49	25
R03	DRUGS FOR OBSTRUCTIVE AIRWAY DISEASES	73	41	32	57	27
S01	OPHTHALMOLOGICALS	70	48	22	61	27
N03	ANTIEPILEPTICS	64	38	26	55	28
M04	ANTIGOUT PREPARATIONS	62	5	57	66	30
C03	DIURETICS	55	28	27	73	30
